# Diminishing Risk for Age-Related Macular Degeneration with Nutrition: A Current View

**DOI:** 10.3390/nu5072405

**Published:** 2013-07-02

**Authors:** Molly Schleicher, Karen Weikel, Caren Garber, Allen Taylor

**Affiliations:** 1 Laboratory for Nutrition and Vision Research, JM USDA Human Nutrition Research Center on Aging Tufts University, 711 Washington St, Boston, MA 02111, USA; E-Mails: molly.schleicher@tufts.edu (M.S.); caren.garber@tufts.edu (C.G.); 2 Boston Medical Center, 650 Albany Street, Boston, MA 02118, USA; E-Mail: karen.weikel@bmc.org

**Keywords:** AMD, antioxidants, carotenoids, nutrition, glycemic index, aging

## Abstract

Age-related macular degeneration (AMD) is the leading cause of blindness in the elderly. Clinical hallmarks of AMD are observed in one third of the elderly in industrialized countries. Preventative interventions through dietary modification are attractive strategies, because they are more affordable than clinical therapies, do not require specialists for administration and many studies suggest a benefit of micro- and macro-nutrients with respect to AMD with few, if any, adverse effects. The goal of this review is to provide information from recent literature on the value of various nutrients, particularly omega-3 fatty acids, lower glycemic index diets and, perhaps, some carotenoids, with regard to diminishing risk for onset or progression of AMD. Results from the upcoming Age-Related Eye Disease Study (AREDS) II intervention trial should be particularly informative.

## 1. Introduction

Age-related macular degeneration (AMD) is the leading cause of blindness worldwide, affecting 30–50 million people. AMD affects over two million individuals of all ages and races in the United States and 11% of those above the age of 80 [1]. Globally, costs associated with AMD are over $340 billion USD, and the majority of AMD patients are not eligible for clinical treatments [2,3,4]. Along with the burgeoning population of elderly, the prevalence of AMD is projected to grow over the next 20–30 years, with over five million individuals being affected by 2050 [5].

AMD is an eye disorder that gradually destroys the macula, the part of the retina with a high density of photoreceptors that is responsible for sharp, high-resolution vision. This part of the retina is able to receive light signals and quickly turn such signals into chemical and then electrical signals that are sent to the brain via the optic nerve. The brain then converts these electrical signals into the images we see. However, if the photoreceptors in the macula are damaged or prematurely shed, as in AMD, the central field of vision is distorted or lost [5].

Photoreceptors are exposed to extensive oxidative stress in the form of light and oxygen [6]. As a result, the outer 10% of photoreceptor segments are shed each night. These must be engulfed, degraded and the debris removed by the retinal pigment epithelium (RPE), which lies posterior to the photoreceptors [7]. Since one RPE cell services 30 photoreceptors, the RPE has among the highest degradative burdens in the body. In addition, the RPE is involved in maintaining the nutriture of the photoreceptors. Since photoreceptors do not have their own blood supply, it is crucial for nutrients from the choroidal blood supply to cross Bruch’s membrane and enter the RPE and photoreceptors [6,8,9]. Adequate nutritional support to the RPE also facilitates efficient turnover of photoreceptors.

The combination of inadequate nutrition and the inability to properly degrade and dispose of cellular debris may contribute to the formation of deposits in the RPE-Bruch’s membrane region. Basal laminar deposits accumulate between the RPE basement membrane and the RPE plasma membrane [10]. These are thought to precede the formation of drusen, which are established clinical indicators for early AMD [11,12,13,14]. Drusen are often found between the RPE and the choroid and contain a variety of lipids and proteins, including ubiquitin and advanced glycation end products, as well as inflammatory mediators [11].

There are two forms of AMD, commonly referred to as dry and wet. Approximately 90% of AMD patients in the US have dry AMD [5]. Dry AMD has three stages, early AMD, intermediate AMD and advanced AMD, which are characterized in part by the size and number of drusen [5]. Early AMD is indicated by small (<63 μm) and/or a few medium-sized (<125 μm) drusen. Intermediate AMD is indicated by many medium-sized or one or more large-sized drusen. Advanced dry AMD, also known as geographic atrophy, is diagnosed when there is focal, round depigmentation with sharp margins and, in some cases, visible choroidal vessels, as well [15]. In addition, there are often large and abundant drusen, as well as RPE and photoreceptor death in the macula. Consequently, patients with geographic atrophy experience significant vision loss.

Approximately 10% of AMD patients in the US have the more visually debilitating neovascular, or wet, form of AMD [5]. Neovascular AMD is manifested by formation of exudates and/or neovascularization of the retina. The latter is characterized by the development of aberrant blood vessels, originating from the choroid, that penetrate Bruch’s membrane, causing damage to the RPE and overlying photoreceptors. These aberrant vessels are prone to leak, thus the designation “wet AMD”. Such bleeding can cause the macula to swell and bulge, causing straight lines to appear curved [16].

Risk factors for AMD include age, gender, race, family history, genetics, weight and smoking. As individuals age, the risk for AMD increases [5]. Gender also appears to influence risk for AMD, with women having a slightly higher risk for AMD than men. Non-Hispanic blacks have less risk for non-exudative AMD at age 80 than Caucasians, and Asians have a higher rate of non-exudative AMD at age 60 than Caucasians [17]. Genetic analyses have shown that a primary relative who has AMD can increase one’s own risk of the disease. Several single nucleotide polymorphisms have been associated with AMD risk in certain populations, with the most widely known polymorphism found on the Complement Factor H gene [18,19,20,21,22]. Obesity is another significant, yet modifiable risk factor, and it has been shown that a 3% reduction in the waist-to-hip ratio decreases risk for AMD by 20% [23]. The most consistently reported risk factor for AMD is smoking, as it increases the risk for AMD up to seven-fold [3].

Currently, there are no therapies to treat dry AMD [24]. There are available treatments for some neovascular AMD patients. However, they are used only after the patients have lost some vision. Clearly, there is a critical need for preventative measures against AMD.

## 2. Reader’s Guide

There have been dozens of studies during the past 40 years. The following sections summarize data that relate relationships between particular nutrients and risk for AMD. Readers are referred to an exhaustive review by Weikel *et al.* [25] for a complete summary in text of the data collected up to 2011 regarding risk for onset or progress of AMD and intake, blood levels or supplement use of specific nutrients. 

For clarity, this review will only use the term AMD, as AMD and age-related maculopathy (ARM) are used interchangeably. Furthermore, those studies that used the term “exudative AMD” will be described here as “neovascular”, since both terms refer to the same condition. Those studies which used the term “severe AMD” will be described here as “late AMD”, as per the majority of studies. Often, this refers to geographic atrophy or exudative, wet AMD.

Data in the figures is organized by type of exposure (intake, plasma level, *etc.*) and outcome measure (early AMD, late AMD, *etc.*). We further divide our discussion by study design, because there are different limitations inherent in each type of study. For each of the nutrients, the figures present risk for “any” type of AMD, early AMD indicators, early AMD, late AMD, followed by geographic atrophy and neovascular AMD, if these specific types of late AMD were analyzed. Data regarding “risk” are separated from data regarding “risk for progression”. Information about the latter is crucial, because it addresses the opportunity to delay early AMD from progressing to vision-compromising disease. In order to present a comprehensive view, results of studies that found beneficial associations of a particular nutrient are reported in the figures with studies that found null associations and studies that found harmful associations of that same nutrient on the same outcome. Data from studies that do not indicate odds or hazard ratios are referenced in the text, but not indicated in the figures.

## 3. Dietary Carbohydrate

Most of the research investigating the role of carbohydrates in AMD risk relates to glycemic index. Glycemic index is the measure of the ability of 50 g of a certain food to raise blood glucose levels, relative to the ability of 50 g of a standard food (e.g., glucose) to raise blood glucose levels [26]. High glycemic foods result in higher levels of glucose in the blood within two hours of consumption. All epidemiologic data published to date indicates that consuming higher glycemic index foods is associated with a greater risk for AMD or AMD progression [27,28].

**Figure 1:**
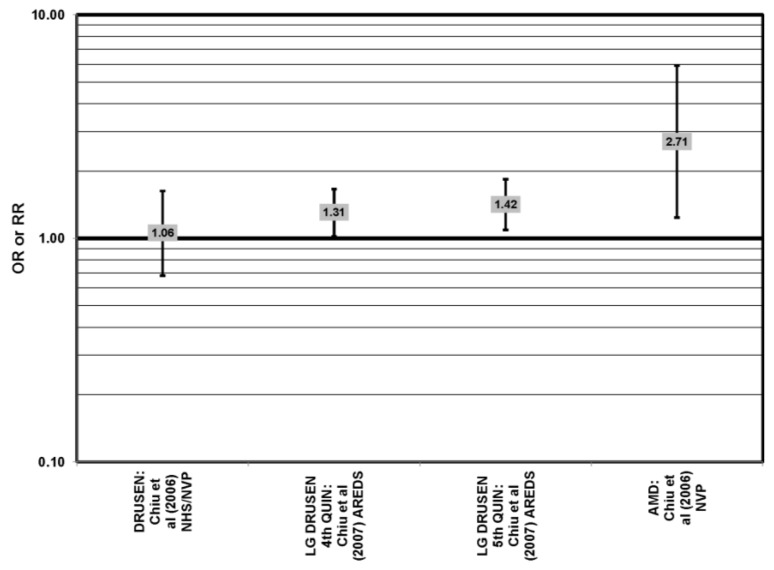
Odds or risk ratio for early age-related macular degeneration (AMD) indicators (DRUSEN), late AMD indicators (LG DRUSEN) or AMD with intake of a high glycemic index diet: cross-sectional studies.

Cross-sectional analysis of baseline Age-Related Eye Disease Study (AREDS) data found that compared to those with dietary glycemic indexes in the first quintile, those with intakes in the fourth (OR (odds ratio) = 1.31; 95% CI (confidence interval): 1.02, 1.66) or fifth quintile (OR = 1.42; 95% CI: 1.09, 1.84) were at an increased risk for the appearance of large drusen [29] (Figure 1). There was also a trend for increasing glycemic index with increasing risk for large drusen (*p* = 0.001) [29]. Increasing dietary glycemic index increased risk for neovascular AMD (*p* = 0.005), and there was a statistically significant trend of increasing dietary glycemic index with advancement of AMD stage (*p* < 0.001) [29]. These observations corroborated findings from a cross-sectional analysis of the Nutrition and Vision Project (NVP), which found that after multivariate adjustment, those who had dietary glycemic indices in the highest tertile had an increased risk for AMD compared to the lowest tertile (OR = 2.71; 95% CI:1.24,5.93; *p* = 0.01, for trend) (Figure 1). Glycemic index did not affect risk for drusen in this population [30] (Figure 1).

**Figure 2:**
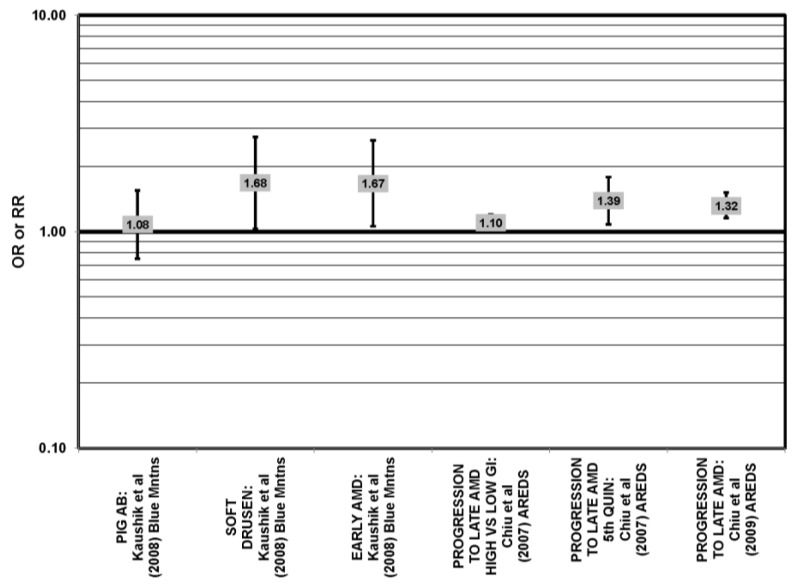
Odds or risk ratio for early AMD indicators (PIG AB), late AMD indicators (SOFT DRUSEN), early AMD, or progression to late AMD, with intake of a high glycemic index diet: prospective studies.

Prospective studies also indicate that higher glycemic index foods increase risk for AMD. In AREDS, risk of progression of AMD over an eight-year period was higher in those with a higher glycemic index diet (RR (relative risk)= 1.10; 95% CI: 1.00, 1.20; *p* = 0.047) [31] (Figure 2). Those at later stages of the disease had a greater risk of progression on the higher glycemic index diet (*p* < 0.001), and compared to those with the lowest quintile of dietary glycemic index, those in the highest quintile of dietary glycemic index had a 39% higher risk of progressing to advanced AMD (95% CI: 1.08, 1.79) [31] (Figure 2). The authors calculated that 20% of the prevalent AMD cases would have been eliminated if participants consumed a diet with a glycemic index below the median and predicted that by changing the dietary glycemic index only slightly, approximately 100,000 cases of AMD would be avoided in five years [29]. Additional analyses including data regarding intake of eicosapentaenoic acid (EPA) and docosahexaenoic acid (DHA) from 2924 AREDS participants revealed that consumption of high glycemic index diets increased the progression to advanced AMD by 32% (95% CI: 1.16, 1.52), and those who consumed a low glycemic index diet along with a high consumption of DHA were at even lower risk for progression to advanced AMD (*p* < 0.001) [32] (Figure 2).

**Figure 3:**
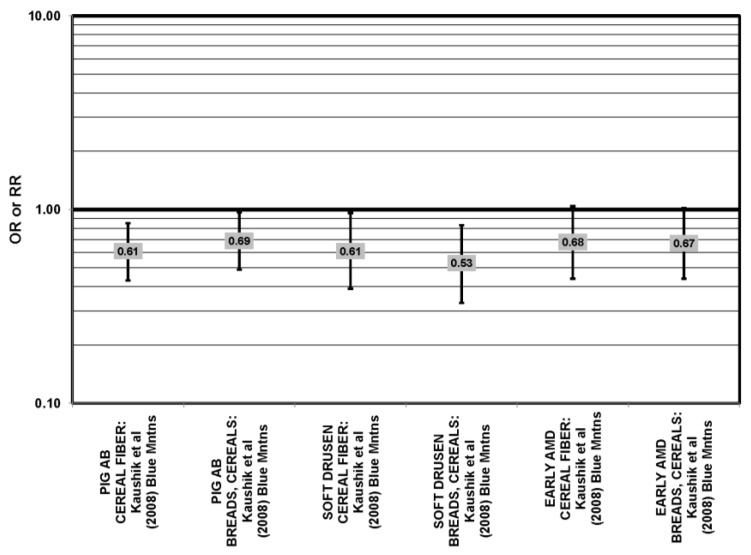
Odds ratio for early AMD indicators (PIG AB), late AMD indicators (SOFT DRUSEN), or early AMD; high *vs.* low intake of low glycemic index foods: prospective study.

**Figure 4:**
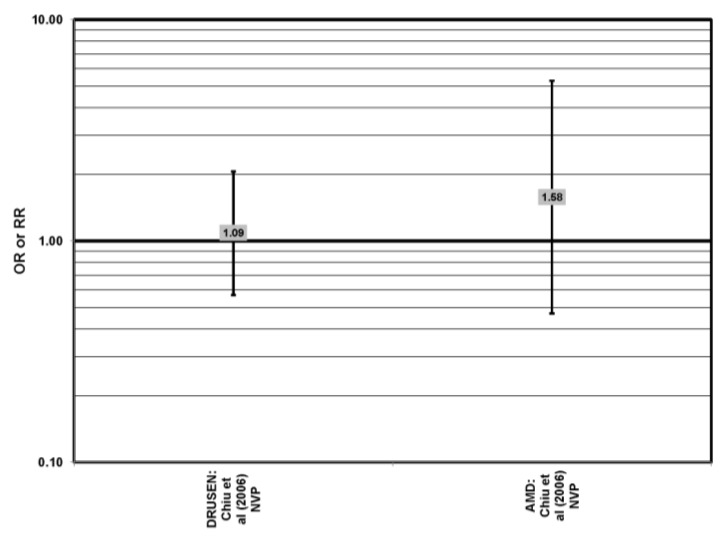
Odds or risk ratio for early AMD indicators (DRUSEN) or AMD; high *vs.* low intake of total carbohydrates: cross-sectional studies.

The Blue Mountains Eye Study corroborated the data from the Nurses’ Health Study (NHS) and AREDS, showing that among 3654 participants 10 years after baseline, those with a dietary glycemic index in the highest quartile were at an increased risk for early AMD compared to those in the lowest quartile, after adjusting for age, sex, BMI (body mass index) smoking, blood pressure, history of cardiovascular disease and vegetable, fruit and fat intake (RR = 1.67; 95% CI: 1.06, 2.64; *p* = 0.04, for trend) (Figure 2). A significant trend of decreasing risk for early AMD with increased consumption of cereal fiber (*p* = 0.05) and breads and grains (*p* = 0.03) was found (Figure 3). Those consuming the highest amounts of cereal fiber, breads and grains had a reduced risk of soft drusen (RR = 0.61; 95% CI: 0.39, 0.96; *p* = 0.01, for trend) and pigment abnormalities (RR = 0.61; 95% CI: 0.43, 0.85; *p* = 0.04, for trend) (Figure 3). Comparison of the highest to lowest quartile of glycemic index also showed an increased risk for soft drusen over 10 years (RR = 1.68; 95% CI: 1.03, 2.74; *p* = 0.04, for trend) (Figure 2). In general, measures of the amount of carbohydrate were not associated with risk for early or late AMD [33] (Figure 4).

Three cohorts have examined the role of carbohydrates in AMD risk. Albeit a limited number of cohorts, they were large cohorts, and the evidence indicates that consumption of carbohydrates of a low glycemic index appears to lower risk for AMD and AMD progression. In comparison, total carbohydrate intake does not appear to be related to risk for or progression of AMD. Controlled laboratory studies have corroborated the data and propose the pathophysiologic mechanisms of the relationship between glycemic index and AMD [34,35]. These data indicate that glycoxidative stress results in accumulation of elevated levels of intracellular glycated proteins and compromised protein editing capacities. This leads to a viscous cycle of glycative damage, diminished proteolytic capacity, accumulation of glycated proteins, cytotoxicity and tissue dysfunction [25].

The above findings are consistent with data supporting consumption of lower glycemic index diets to decrease risk for obesity, a risk factor for AMD [36]. Several intervention studies have found that energy-restricted diets based on low glycemic index foods contribute to greater weight loss than calorically equivalent diets based on high glycemic index foods [37]. Taken together, these observational data encourage use of low glycemic index diets to lower the risk for AMD and AMD progression.

## 4. Dietary Fats and Fish

### 4.1. Omega-3 and Omega-6 Fatty Acids

Increased intake of omega-3 fatty acids, especially long-chain omega-3 fatty acids, such as DHA and EPA, found in fish, has been associated with amelioration of a number of chronic diseases, including AMD [38,39].

**Figure 5:**
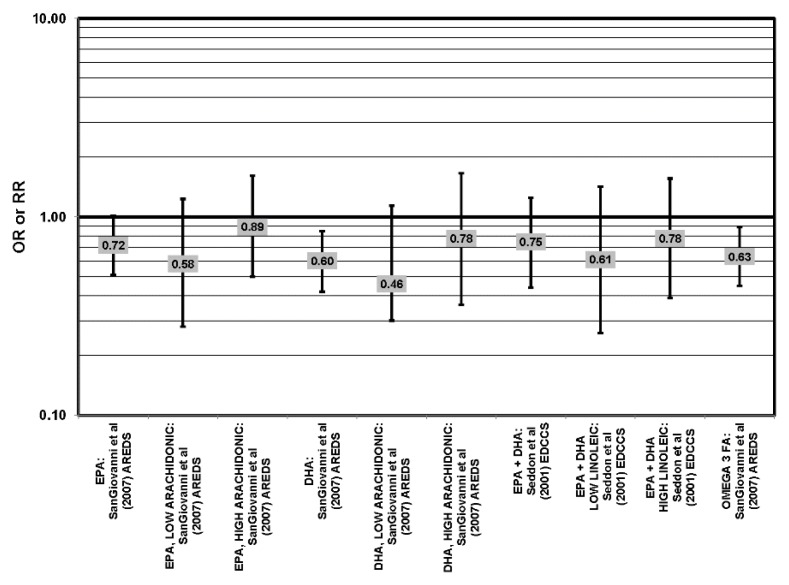
Neovascular AMD odds or risk ratio, high *vs.* low intake of omega-3 fatty acids: retrospective and cross-sectional studies.

The Eye Disease Case Control Study (EDCC), which consisted of 349 cases and 504 controls, found that, in subjects with a low linoleic acid (omega-6 fatty acid) intake, there was a trend of retinal protection in those with higher intake of omega-3 fatty acids (*p* = 0.05). Without adjusting for omega-6 intake, this trend became non-significant (*p* = 0.29). This trend suggests that omega-6 and omega-3 fatty acids may be in a state of metabolic competition. However, while biochemical studies continue to suggest competition, further epidemiologic analysis did not support such competition. When comparing those with the highest and lowest EPA and DHA consumption, EPA and DHA did not confer significant protection from neovascular AMD before adjusting for linoleic acid intake (OR = 0.75; 95% CI: 0.44, 1.25) or in those with low (OR = 0.61; 95% CI: 0.26, 1.42) or high linoleic acid intake (OR = 0.78; 95% CI: 0.39, 1.56) [40] (Figure 5).

**Figure 6:**
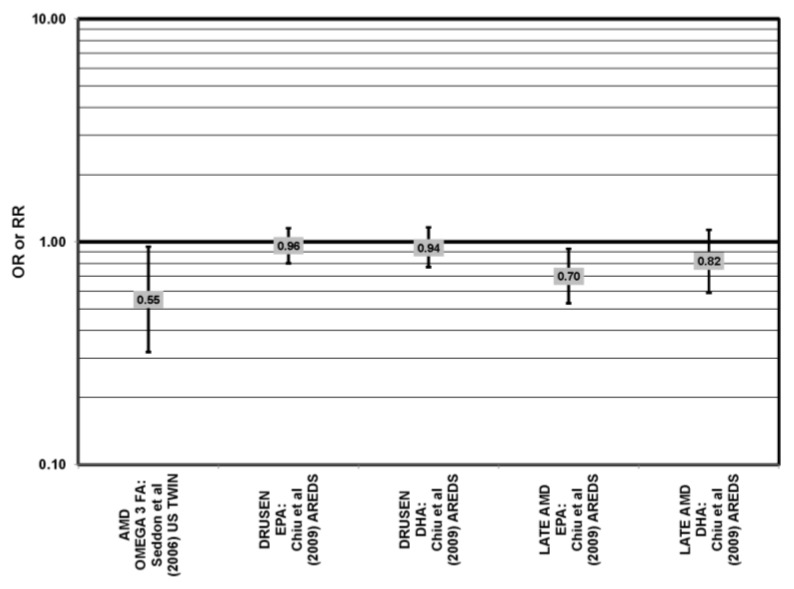
Odds or risk ratio for early AMD indicators (DRUSEN), early AMD or LATE AMD and OR AMD; high *vs.* low intake of omega-3 fatty acids: retrospective and cross-sectional studies.

Strong evidence for a beneficial role of omega-3 fatty acids in eye health is found in two cross-sectional studies, the US Twin Study of Age-Related Macular Degeneration (“US Twin”) and AREDS. The US Twin study found that compared to those consuming the least amount of omega-3 fatty acids, those consuming the highest amount had a reduced risk for any stage of AMD (OR = 0.55; 95% CI: 0.32, 0.95). This association was driven mostly by those with a low linoleic and omega-6 fatty acid intake (*p* < 0.001), as the association disappeared in those with an intake of linoleic acid above the median [41] (Figure 6). Analysis of the baseline data from 4519 participants in AREDS revealed that compared to those in the lowest quintile of intake, those in the highest quintile of intake for EPA (OR = 0.72; 95% CI: 0.51, 1.01; *p* < 0.05), DHA (OR = 0.60; 95% CI: 0.42, 0.85) and total long-chain omega-3 fatty acids (OR = 0.63; 95% CI: 0.45, 0.89) were at a reduced risk for neovascular AMD [42] (Figure 5). However, sub-group analysis of the population found that the reduction of risk associated with high consumption of EPA and DHA became non-significant when the cohort was separated by intake of arachidonic acid, another omega-6 fatty acid [42] (Figure 5).

**Figure 7:**
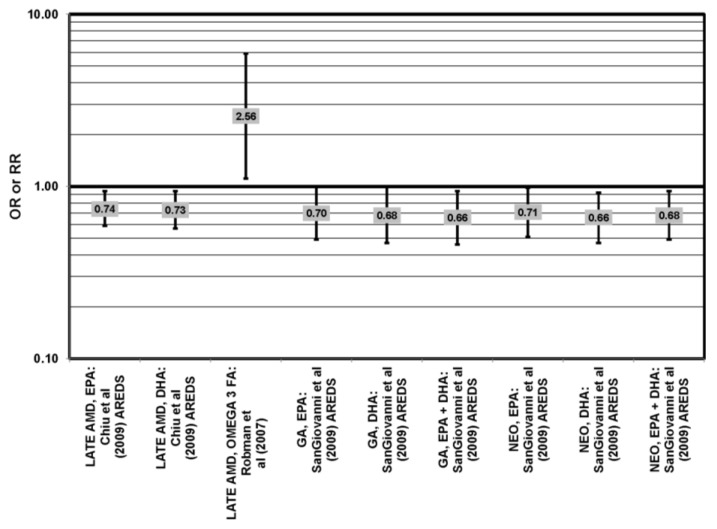
Odds or risk ratio for progression to late AMD, geographic atrophy (GA) or neovascular AMD (NEO); high *vs.* low intake of omega-3 fatty acids: prospective studies.

**Figure 8:**
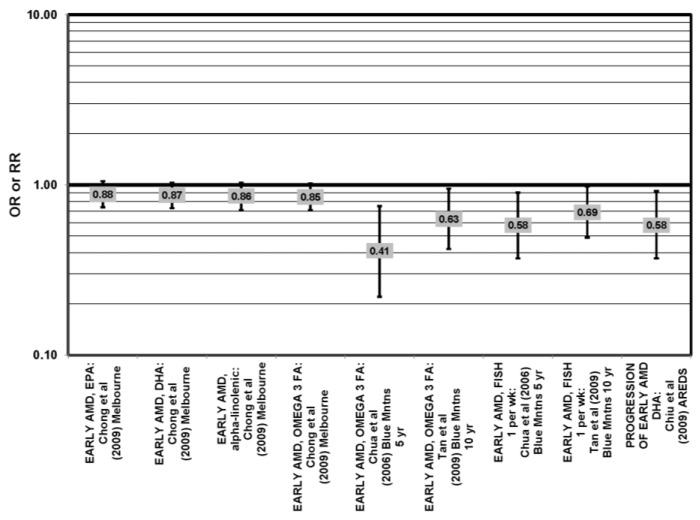
Odds or risk ratio for early AMD or progression to early AMD; high *vs.* low intake of omega-3 fatty acids: prospective studies.

**Figure 9:**
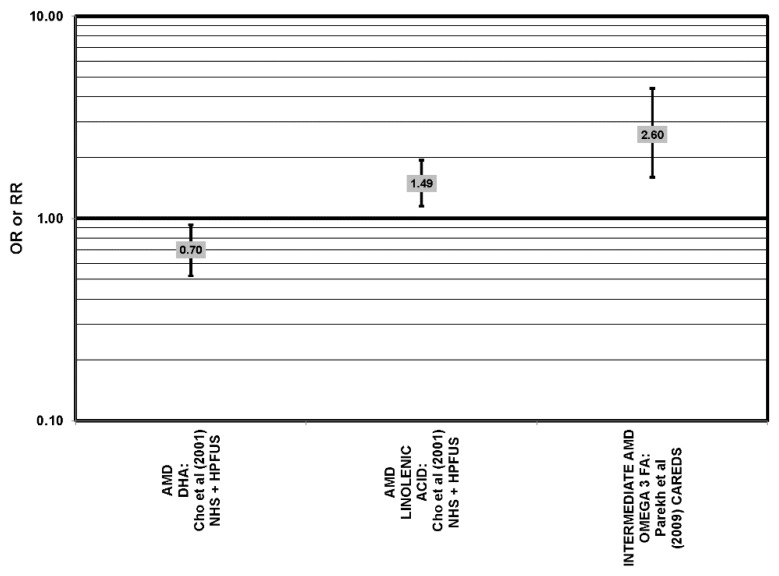
Odds or risk ratio for AMD or intermediate AMD; high *vs.* low intake of omega-3 fatty acids: prospective studies.

**Figure 10:**
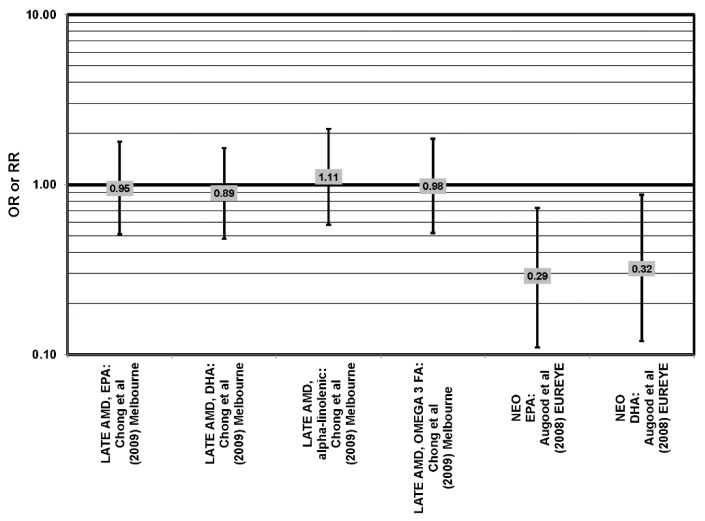
Odds or risk ratio for late, neo AMD; high *vs.* low intake of omega-3 fatty acids: prospective studies.

Data from prospective studies support a beneficial role of omega-3 fatty acids. Analysis of 1837 participants of AREDS found that increasing intake of DHA + EPA was associated with a decreased rate of progression to central geographic atrophy over 12 years (*p* = 0.026), and progression to central geographic atrophy was lowest in subjects with intakes in the highest quintiles of intake of DHA (OR = 0.68; 95% CI: 0.47, 0.99), EPA (OR = 0.70; 95% CI: 0.49, 1.00) and DHA + EPA (OR = 0.66; 95% CI: 0.46, 0.94) (Figure 7). Increasing intakes of DHA alone (*p* = 0.001) or DHA + EPA (*p* = 0.032) were also associated with a decrease in risk of progression to neovascular AMD (*p* = 0.001). Analysis of 2,924 AREDS participants revealed that those consuming more than 64 mg/day of DHA, compared to less than 26 mg/day, had a reduced risk for progression to advanced AMD (HR (hazard ratio) = 0.73; 95% CI: 0.57, 0.94) (Figure 7). Those consuming at least 42.3 mg EPA per day, compared to less than 12.7 mg/day, were at a reduced risk for progression to advanced AMD (HR = 0.74; 95% CI: 0.57, 0.94) (Figure 7). Participants who were healthy at baseline benefitted from a high DHA diet, as indicated by reduced progression of early AMD (HR = 0.58; 95% CI: 0.37, 0.92) [32] (Figure 8). Prospective analysis of 38,022 women from the Women’s Health Study observed that women in the highest tertile of DHA intake, compared to those with the lowest DHA intake, had a 38% reduced risk for AMD (RR = 0.62, 95% CI: 0.45, 0.85) (*p* = 0.003 for trend). Similar relationships were found for higher intake of EPA (RR = 0.64, 95% CI: 0.46, 0.88) (*p* for trend = 0.004), and for DHA + EPA ( RR, 0.62, 95% CI: 0.45–0.86) (*p* for trend = 0.03) [43]. A study of over 72,000 participants from the NHS and Health Professionals’ Follow-Up Study (HPFUS) indicated that those with the highest intakes of DHA were at a reduced risk for AMD (RR = 0.70; 95% CI: 0.52, 0.93) [44] (Figure 9). Analysis of 6339 participants from the Melbourne Collaborative Cohort (“Melbourne”) indicated that those consuming the highest amounts of omega-3 fatty acids were at a slightly reduced risk for early AMD (OR = 0.85; 95% CI: 0.71, 1.02; *p* = 0.03, for trend) (Figure 8), but there was no association between particular fatty acids, such as EPA, DHA and alpha-linolenic acid, and early or late AMD [45]. Benefits of omega-3 fatty acids in general were seen in 2454 participants of the Blue Mountains Eye Study. Compared to those with the lowest intakes of omega-3 fatty acids, those with the highest intakes were at reduced risk for incidence of early AMD (RR = 0.63; 95% CI: 0.42, 0.95) [46] (Figure 8) and such findings were corroborated in follow-up analysis (OR = 0.41; 95% CI: 0.22, 0.75) [47] (Figure 8). Finally, data from a European prospective cohort, EUREYE (The European Eye Study), indicated that among 2275 participants, those with consumption levels of DHA (OR = 0.32; 95% CI: 0.12, 0.87) and EPA (OR = 0.29, 95% CI: 0.11, 0.73) in the highest quartile had a reduced risk for neovascular AMD [48] (Figure 10).

In one intervention trial, supplementation of 12 women with 800 mg DHA per day for four months significantly increased macular pigment optical density (MPOD), a surrogate marker for retina health, after two and four months of supplementation (*p* < 0.001). Surprisingly, women who also received 12 mg/day of lutein with the DHA did not experience as great an increase in MPOD at two months, but had similar density at four months [49]. The optimism that is engendered by the robust epidemiologic record is qualified somewhat by results from the Nutritional AMD Treatment 2 Study [50]. Patients were given either 840 mg/day DHA and 270 mg/day EPA or placebo for three years. For patients that showed the highest levels of EPA + DHA in red blood cell membranes, there was a 68% lower risk for chorodial neovascularization (CNV), but not for other indicators of AMD status. For the full cohort of patients, supplementation with DHA and EPA was without effect relative to placebo. 

The possible connection between omega-6/omega-3 fatty acid ratio and development and progression of AMD has been investigated. Mance *et al.* divided 125 AMD patients into five groups according to the Clinical Age-Related Maculopathy Staging System and measured intake of dietary fatty acids using the validated food frequency questionnaire. A statistically significant difference was found between the omega-6/omega-3 ratio in neovascular AMD compared to all other groups, with the ratio of 11:1 found in the stage 5 group. Additionally, the stage 4 group had a statistically significant difference in the ratio compared to stages 1, 2 and 3, with the ratio in the first three groups at about 7–7.5:1 [51]. Christen *et al.* also found that the ratio of omega-6/omega-3 fatty acids was directly associated with risk for AMD in a group of 38,022 women enrolled in the Women’s Health Study [43].

Despite these studies showing a beneficial role of omega-3 fatty acids in eye health, cross-sectional analysis of 4003 AREDS participants showed no association between DHA intake (OR = 0.94; 95% CI: 0.77, 1.16), EPA intake (OR = 0.96; 95% CI: 0.84, 1.15) and late AMD (OR = 0.82; 95% CI: 0.59, 1.13). There was also no association between intake of either DHA or EPA and risk for drusen (OR = 0.94; 95% CI: 0.77, 1.16) (OR = 0.96; 95% CI: 0.84, 1.15). Although those in the third quartile of EPA intake had 30% less risk for late AMD compared to those with the lowest intakes (95% CI: 0.53, 0.93), this association was lost at higher levels of EPA intake [52]. In the Melbourne cohort, there was also no association between intake of EPA, DHA or alpha-linolenic acid (another omega-3 fatty acid) and risk for early or late AMD [45] (Figure 8, Figure 9). 

**Figure 11:**
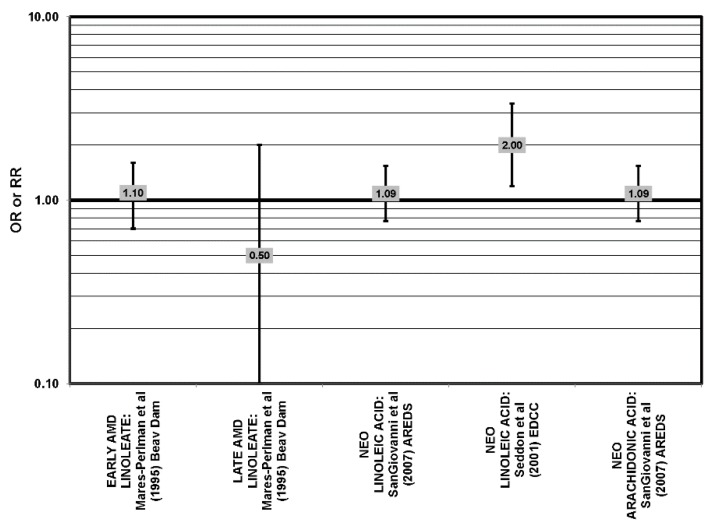
Odds or risk ratio for early AMD, late AMD or neovascular AMD (NEO); high *vs.* low intake of omega-6 fatty acids: retrospective and cross-sectional studies.

**Figure 12:**
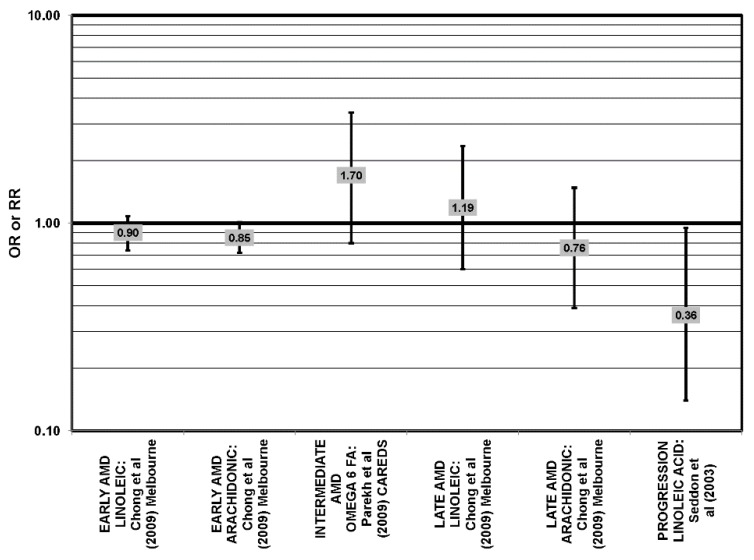
Odds or risk ratio for intermediate AMD, neovascular AMD (NEO) or AMD progression; high *vs.* low intake of omega-6 fatty acids: prospective studies.

With the exception of the EDCC and NHS + HPFUS, most retrospective, cross-sectional and prospective studies found no relationship between omega-6 fatty acid intake (independent of omega-3 fatty acids) and AMD (Figure 11, Figure 12) [42,45,53,54,55]. The EDCC did indicate that high intakes of linoleic acid appeared to increase risk for neovascular AMD (OR = 2.00; 95% CI: 1.19, 3.37) [40] (Figure 11). Additionally, the NHS + HPFUS also found that those with the highest intakes of linolenic acid had increased risk for any stage AMD [44]. The Carotenoids in Age Related Eye Disease Study (CAREDS) found that those consuming the highest amounts of omega-3 fatty acids were at higher risk for intermediate AMD [54]. An additional study found that over seven years, early AMD patients with intakes of omega-3 fatty acids in the highest quartile were at increased risk for AMD. However, this association was lost with a more rigid definition of AMD [55] (Figure 9, Figure 10).

### 4.2. Fish Intake

**Figure 13:**
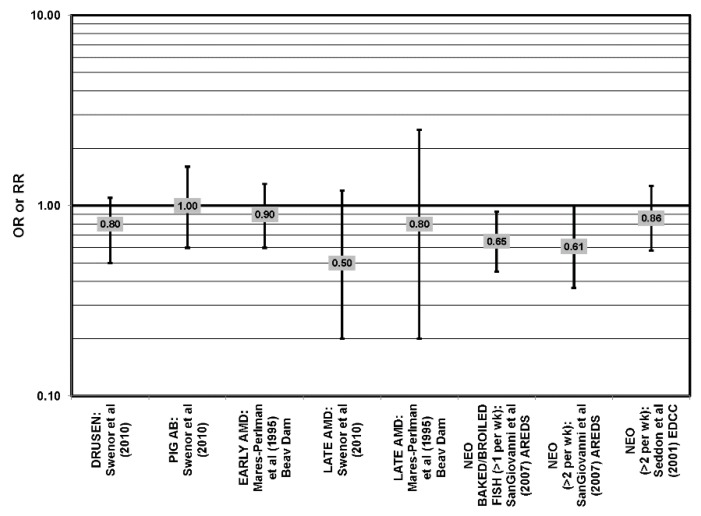
Relationship between early indicators of AMD (DRUSEN, PIG AB), early, late or neovascular (NEO) AMD and fish intake: retrospective and cross-sectional studies.

The effect of fish on risk for AMD is of great interest, as fish is a common dietary source of omega-3 fatty acids [56]. Cross-sectional analyses of AREDS participants indicated that consumption of at least two servings of fish per week was associated with decreased risk for neovascular AMD compared to zero servings per week (OR = 0.61; 95% CI: 0.37, 1.00; *p* = 0.01 for trend). Consumption of more than one serving of baked or broiled fish was associated with a decreased risk of neovascular AMD (OR = 0.65; 95% CI: 0.45, 0.93; *p* = 0.02, for trend) [42] (Figure 13).

**Figure 14:**
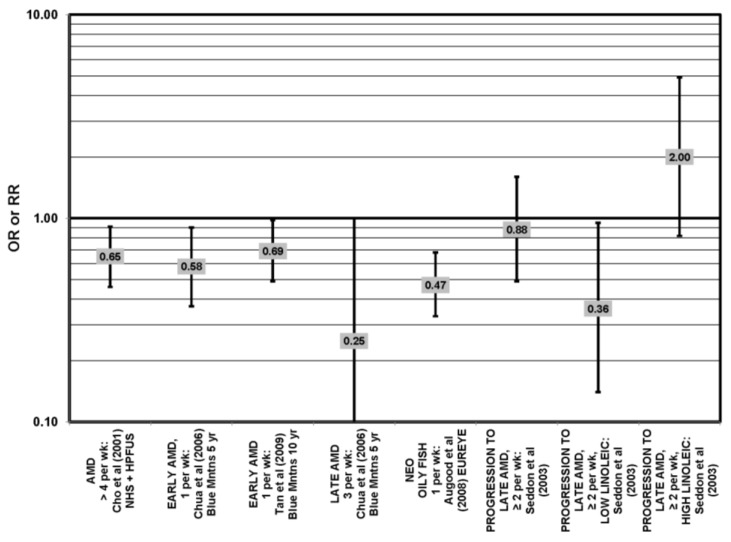
Relationship between early indicators of AMD (DRUSEN, PIG AB), early, late or neovascular (NEO) AMD and fish intake: prospective studies.

Two prospective studies analyzing the effect of fish intake on risk for AMD corroborated the observation of competition between omega-3 and omega-6 fatty acids in modulating eye health that was observed in the EDCC analysis [40]. Data from the Blue Mountains Eye Study showed that among 2454 participants with low linoleic acid (omega-6) consumption, one serving of fish/week was associated with a reduced risk of incident early AMD (OR = 0.69; 95% CI: 0.49, 0.98) ten years after baseline [46] (Figure 14). Another study reported that among those consuming at least two servings of fish/week, those consuming below the median amount of linoleic acid were at a slightly reduced risk for progression to advanced AMD compared to those consuming above the median (RR = 0.36; 95% CI: 0.14, 0.95; *p* = 0.045, for trend) (Figure 14). The beneficial associations of fish intake were not observed in those with higher intakes of linoleic acid, nor were they observed before adjusting for linoleic acid intake [57] (Figure 14). Although these observations contradict those in the quintile intake analysis of the EDCC, the prospective design of these studies increases the likelihood of a competition between omega-3 fatty acids and linoleic acid [40,46,57]. Additionally, analysis of the Women’s Health Study observed that consumption of one or more servings of fish/week was associated with a 42% lower risk of AMD compared to consumption of less than one serving of fish per month [43].

Additional prospective studies support the role of fish in reducing AMD risk, even without adjusting for omega-6 fatty acid intake. Analysis of the combined NHS + HPFUS indicated that those who consumed more than four servings of fish/wk had a reduced risk for any stage of AMD relative to those consuming less than four servings per week (RR = 0.65; 95% CI: 0.46, 0.91) [44] (Figure 14). Another study reported that consuming fish once a week reduced risk for early AMD by 42% (OR = 0.58; 95% CI: 0.37, 0.90), while consumption of fish three times a week reduced risk for late AMD by 75% (95% CI: 0.06, 1.00) [47] (Figure 14). Data from EUREYE indicated that weekly consumption of oily fish was associated with a reduced risk of neovascular AMD (OR = 0.47; 95% CI: 0.33, 0.68) [48] (Figure 12).

The EDCC, retrospective analysis of the Beaver Dam Eye Study and cross-sectional data from the Blue Mountains Eye Study did not find an association between fish intake and early or late AMD [40,53,58,59] (Figure 13). 

### 4.3. Polyunsaturated Fat and Nut Intake

**Figure 15:**
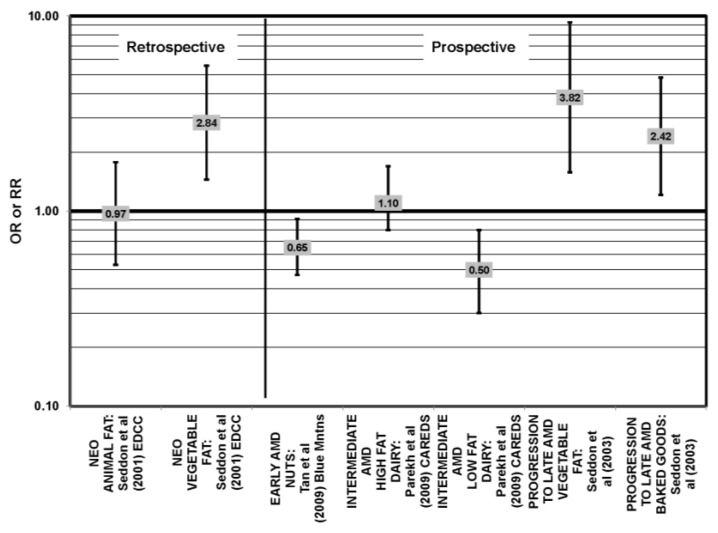
Odds or risk ratio for early AMD, intermediate AMD, progression to late AMD, and neovascular AMD (NEO); high *vs.* low intake of fat-containing foods: retrospective and prospective studies.

**Figure 16:**
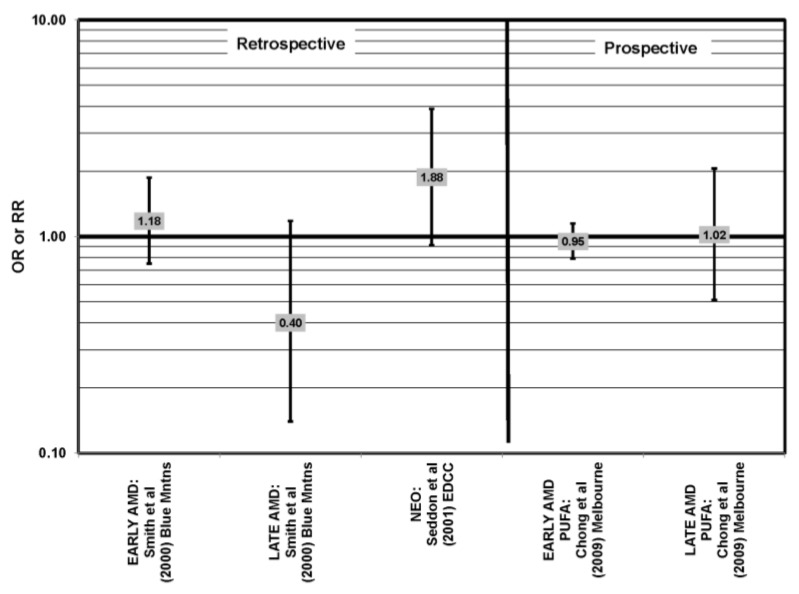
AMD odds or risk ratio; high *vs.* low intake of polyunsaturated fatty acids: retrospective and cross-sectional studies, prospective.

Nuts are a popular source of polyunsaturated fatty acids. In the prospective Blue Mountains Eye Study, it was found that one to two servings of nuts/week was associated with a decreased risk of early AMD (OR = 0.65; 95% CI: 0.47, 0.91) among nonsmokers with low HDL and high intake of beta-carotene [46,56] (Figure 15). All other studies, such as the EDCC, Melbourne Collaborative Cohort, POLANUT (dietary survey study of the Pathologies Oculaires Liées à l’Age study) and cross-sectional analysis of the Blue Mountains Eye Study, did not find a significant association between these types of fatty acids and AMD risk [40,45,55,58] (Figure 16).

### 4.4. Monounsaturated Fatty Acids

**Figure 17:**
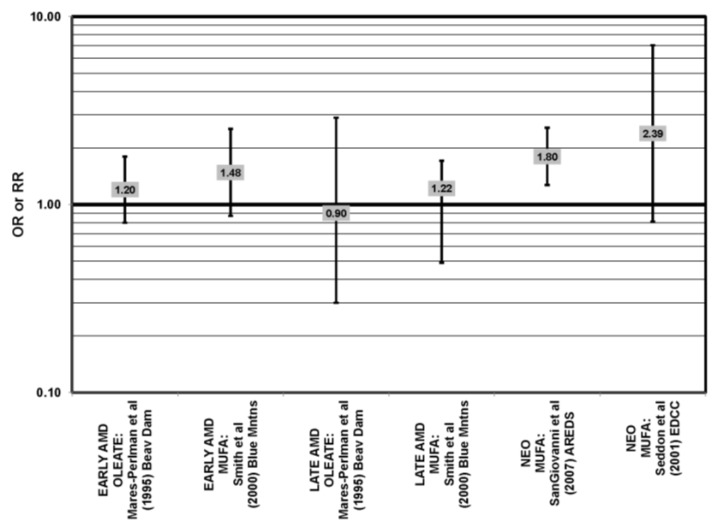
Odds or risk ratio for early AMD, intermediate AMD, late AMD or neovascular AMD (NEO); high *vs.* low intake of monounsaturated fatty acids: retrospective, cross-sectional studies.

**Figure 18:**
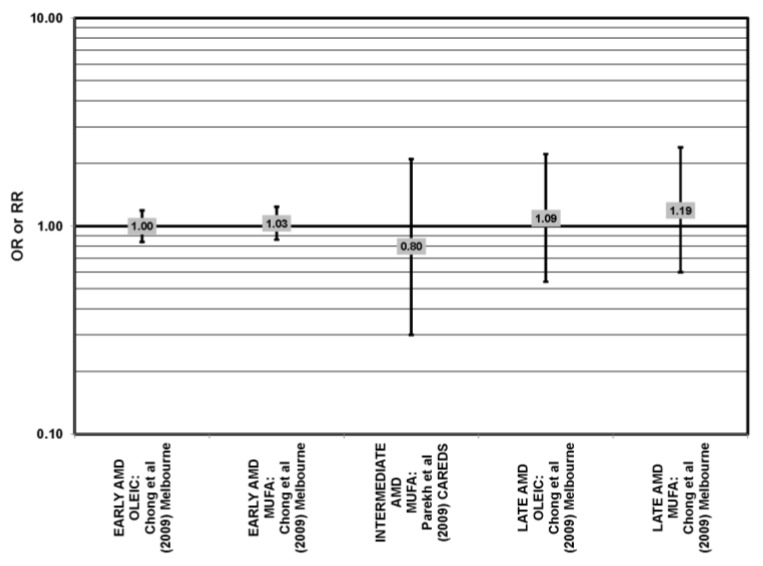
Odds or risk ratio for early AMD, intermediate AMD or late AMD; high *vs.* low intake of monounsaturated fatty acids: prospective studies.

Many studies, including the EDCC, Beaver Dam Eye Study, CAREDS, Melbourne Collaborative Cohort and the POLANUT study, did not find a significant association between consumption of monounsaturated fatty acids, such as oleic acid, and any stage of AMD risk [40,45,53,54,55,60] (Figure 17, Figure 18). Cross-sectional analysis of 3654 subjects from the Blue Mountains Eye Study, and cross-sectional analysis of AREDS found a slightly harmful trend of increasing consumption of monounsaturated fatty acids and increasing risk for early and neovascular AMD (*p* = 0.05) and (OR = 1.80; 95% CI: 1.27, 2.56), respectively. Oleic acid, a commonly consumed monounsaturated fatty acid, was not significantly associated with disease risk in AREDS [42,53,58] (Figure 17).

### 4.5. Saturated Fat

**Figure 19:**
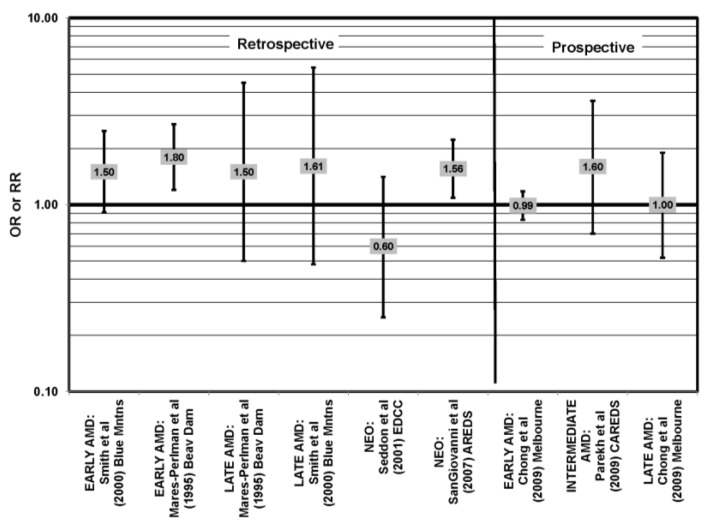
Odds or risk ratio for early AMD, intermediate AMD, late AMD or neovascular AMD (NEO); high *vs.* low intake of saturated fat: retrospective, cross-sectional and prospective studies.

No studies have reported retinal benefits through consumption of saturated fatty acids The EDCC, CAREDS, Blue Mountain Eye Study, Melbourne cohort, POLANUT and Cardiovascular Health and Age Related Maculopathy study did not find an association between saturated fat intake and risk for AMD [40,45,54,55,58,60] (Figure 15, Figure 19). However, analysis of the Beaver Dam Eye study and AREDS showed an association between high saturated fat intake and increased risk for AMD [42,53] (Figure 19).

### 4.6. Trans Fatty Acids

**Figure 20:**
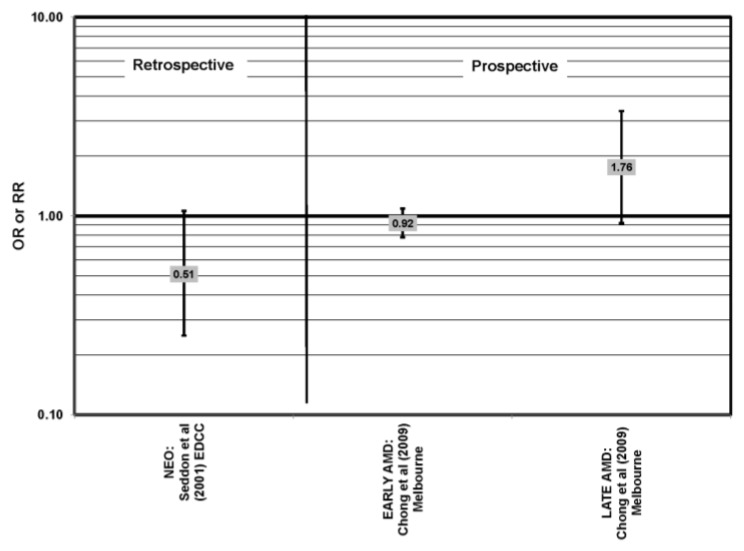
Odds or risk ratio for late AMD or neovascular AMD (NEO); high *vs.* low intake trans-fatty acids: retrospective and prospective studies.

The EDCC and Melbourne Collaborate Cohort analyzed the role of trans-fatty acid intake in AMD risk. Both did not find a significant relationship with early, late or neovascular AMD risk, nor with AMD progression [45,55] (Figure 20).

### 4.7. Cholesterol

**Figure 21:**
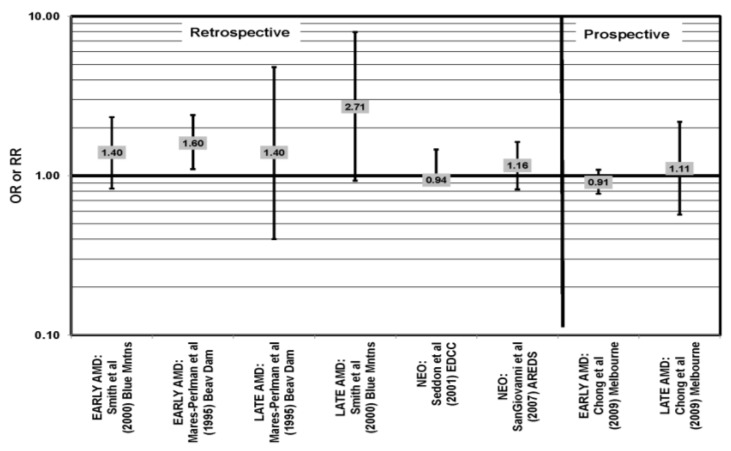
Odds or risk ratio for early AMD, late AMD or neovascular AMD (NEO); high *vs.* low intake of dietary cholesterol: retrospective and cross-sectional studies.

Excessive cholesterol intake is related to poor cardiovascular health and elevated cholesterol levels. Similarly, no studies have found retinal benefits through high cholesterol intake. Cholesterol intake was not found to be associated with neovascular AMD risk in cross-sectional analysis of AREDS, with early AMD in the Blue Mountains Eye Study or with early or late AMD in the Melbourne cohort [40,42,45,58]. The Beaver Dam Eye and Blue Mountains Eye studies found an association of high cholesterol intake with increased risk for early AMD [53,58] (Figure 21).

### 4.8. Total Fat

**Figure 22:**
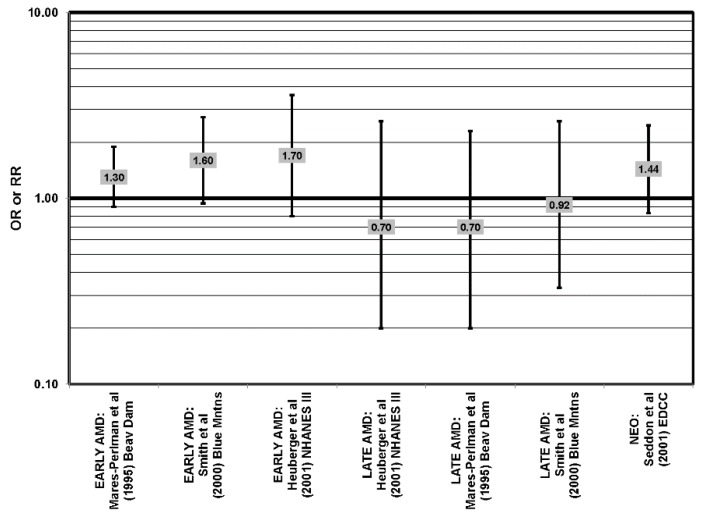
AMD odds or risk ratio; high *vs.* low intake of total fat: retrospective and cross-sectional studies.

**Figure 23:**
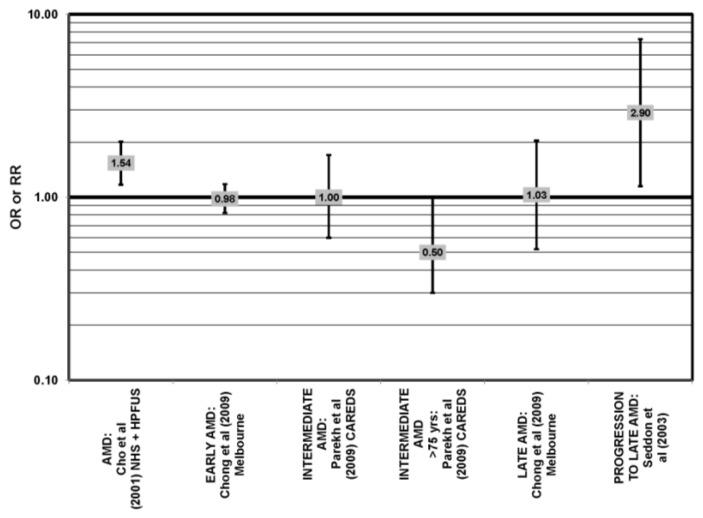
Odds or risk ratio for AMD, intermediate AMD or progression to late AMD; high *vs.* low intake of total fat: prospective studies.

Only CAREDS has found a potential beneficial role of total fat intake. Women in CAREDS over the age of 75 with a high fat intake appeared to be protected from intermediate AMD (OR = 0.50; 95% CI: 0.30, 1.00) [54] (Figure 22). Retrospective analysis of the Beaver Dam Eye study and EDCC found no relationship between total fat intake and risk for neovascular AMD [40,53]. Similarly, cross-sectional analysis of the National Health and Nutrition Education Evaluation Survey (NHANES) and several prospective cohorts did not find a relationship between total fat intake and AMD risk [45,54,55,61]. It was found that those consuming the highest levels of total fat were at increased risk for any stage of AMD, as well as increased risk for AMD progression in the POLANUT study (*p* = 0.007), NHS + HPFUS (95% CI: 1.17, 2.01; *p* = 0.008, for trend) and a study of 261 dry AMD patients (RR = 2.90; 95% CI: 1.15, 7.32; *p* = 0.01, for trend) [57,60] (Figure 23).

### 4.9. Animal versus Vegetable Fats

A few cohorts studied the associations of either animal or vegetable fats on AMD risk. The EDCC did not find an association between animal fat intake and risk for neovascular AMD, but found that compared to those consuming the lowest amounts of vegetable fat, those consuming the highest amounts had an increased risk for neovascular AMD (OR = 2.84; 95% CI: 1.45, 5.57; *p* = 0.006, for trend) [40] (Figure 15). Furthermore, a prospective study of 261 dry AMD patients found that over 4.6 years, compared to those consuming the lowest levels of vegetable fat, those consuming the highest levels were at an increased risk for progression to advanced AMD (RR = 3.82; 95% CI: 1.58, 9.28; *p* = 0.003, for trend) [57] (Figure 15).

### 4.10. Summary

To date, the body of observational epidemiologic data indicates that increased consumption of EPA and DHA reduces risk for neovascular and early AMD. However, such a relationship was not ascertained between consumption of omega-6 fatty acids and risk for AMD in the majority of studies that analyze this relationship. The relation of fish consumption and AMD is not as strong as the DHA and EPA components of fish individually. This suggests that the benefits of omega-3 fatty acids might be attenuated, due to interactions with other components of fish, such as omega-6 fatty acids [25]. In addition, the foods consumed with the fish and potential decreases in consumption of other food groups to compensate for increased fish are difficult to disentangle and may confound the results. The impact of monounsaturated and saturated fat consumption on AMD risk is unclear at this time. This may be due in part to different analytic approaches across studies. Further investigation into relationships between intakes of animal and vegetable fat, specifically trans-fat, as well as cholesterol, and risk for AMD may be warranted.

## 5. Carotenoids

### 5.1. Lutein and Zeaxanthin

**Figure 24:**
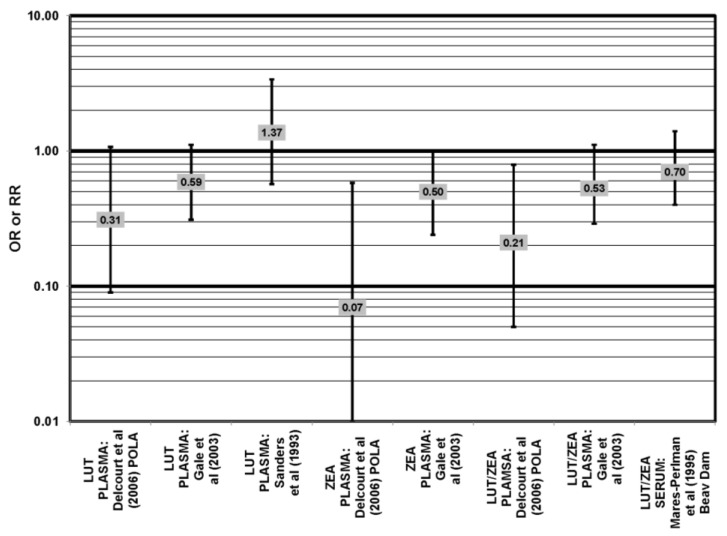
AMD odds or risk ratio; high *vs.* low blood levels of lutein and/or zeaxanthin: retrospective and cross-sectional studies.

**Figure 25:**
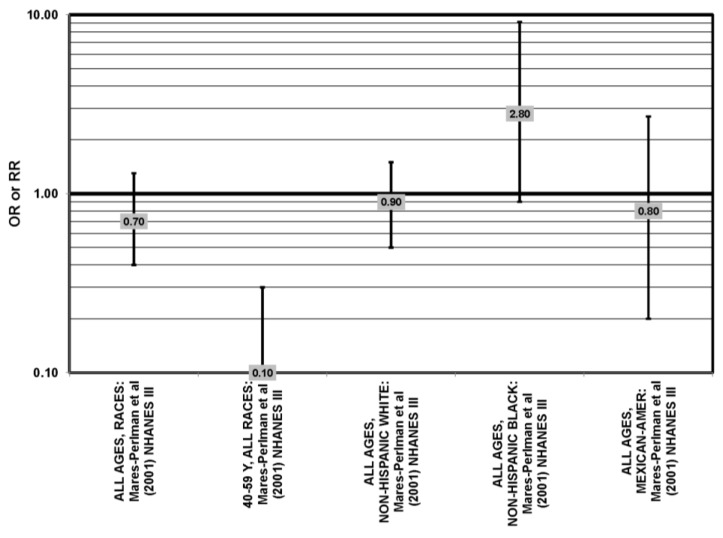
Odds or risk ratio for pigmentary abnormalities; high *vs.* low intake of lutein and zeaxanthin: retrospective and cross-sectional studies.

**Figure 26:**
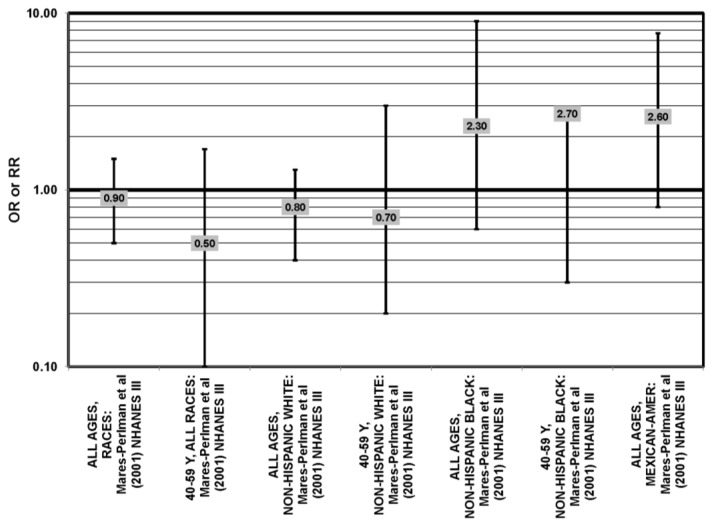
Odds or risk ratio for pigmentary abnormalities; high *vs.* low serum levels of lutein and zeaxanthin: retrospective and cross-sectional studies.

**Figure 27:**
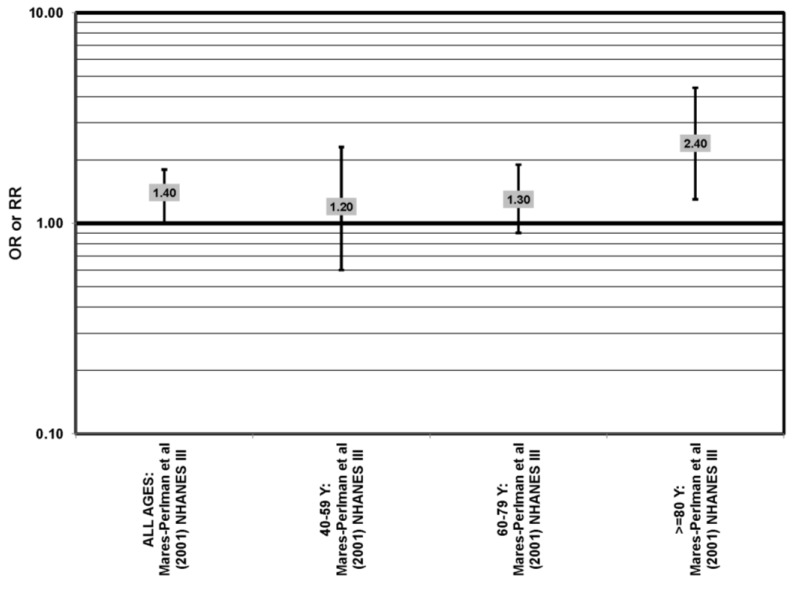
Odds or risk ratio for soft drusen; high *vs.* low intake of lutein and zeaxanthin:retrospective studies.

**Figure 28:**
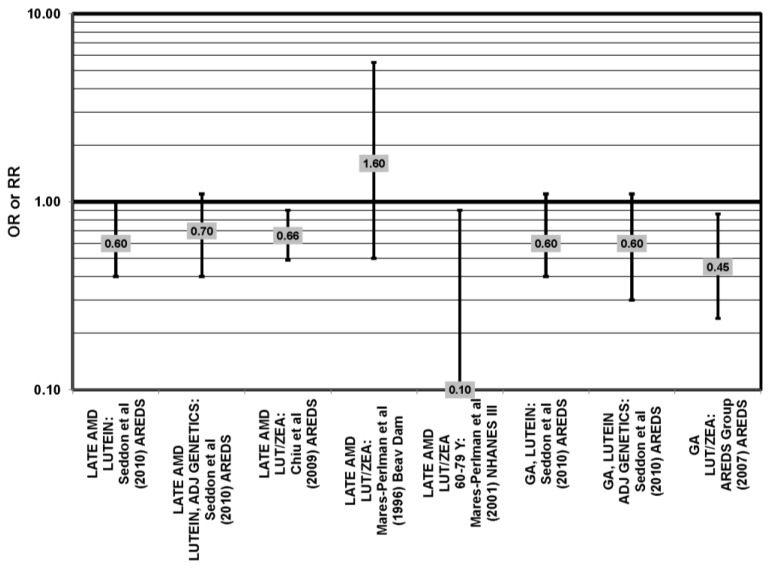
Odds or risk ratio for late AMD or geographic atrophy (GA); high *vs.* low intake of lutein and zeaxanthin from food: retrospective and cross-sectional studies.

**Figure 29:**
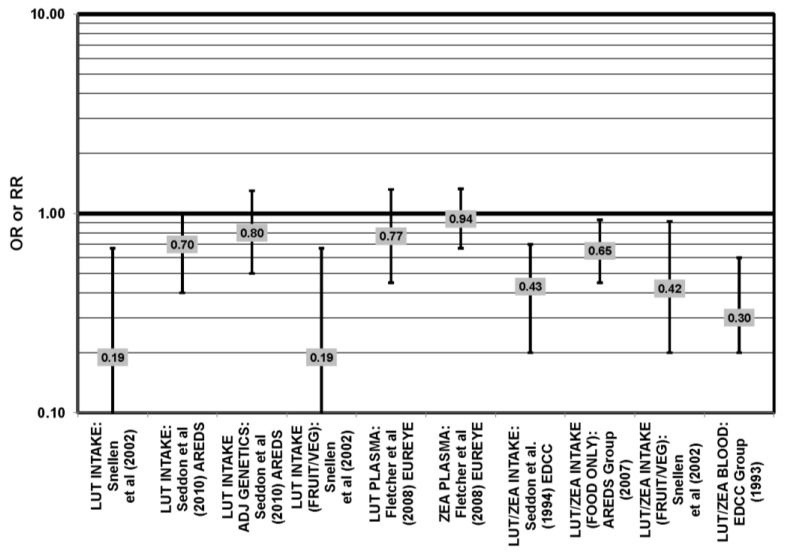
Odds or risk ratio for exudative or neovascular AMD; high *vs.* low intake or blood levels of lutein and/or zeaxanthin: retrospective and cross-sectional studies.

**Figure 30:**
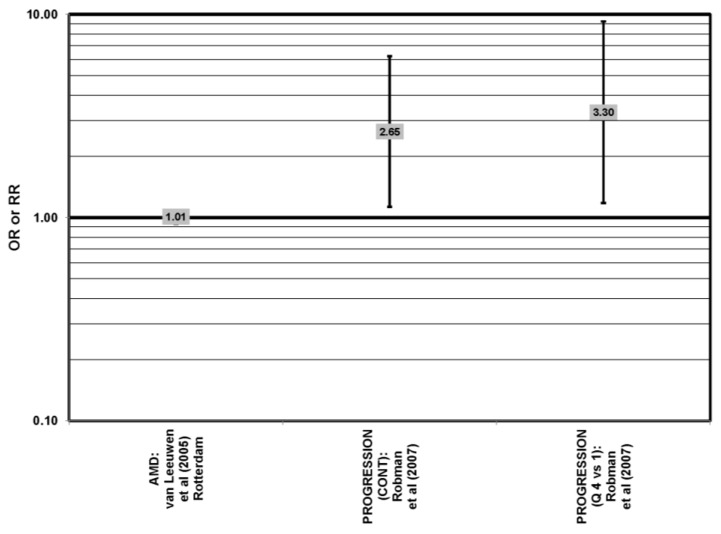
AMD odds or risk ratio; high *vs.* low intake of lutein and zeaxanthin: prospective studies.

**Figure 31:**
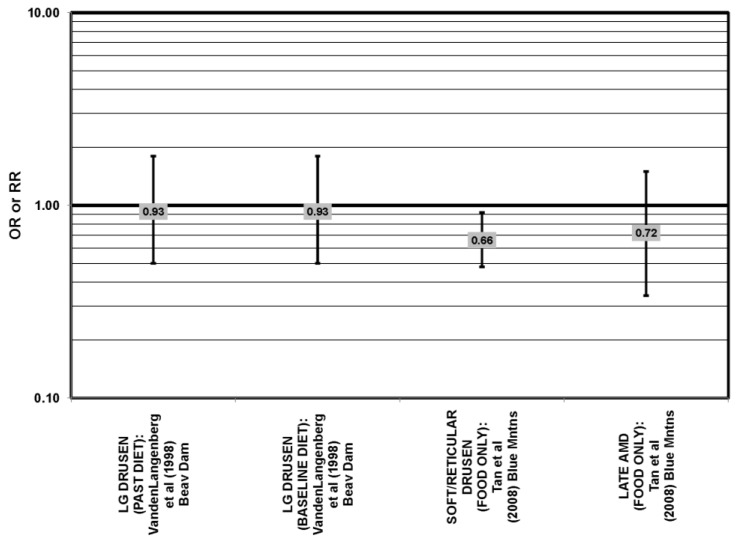
Late AMD odds or risk ratio; high *vs.* low intake of lutein and zeaxanthin: prospective studies.

**Figure 32:**
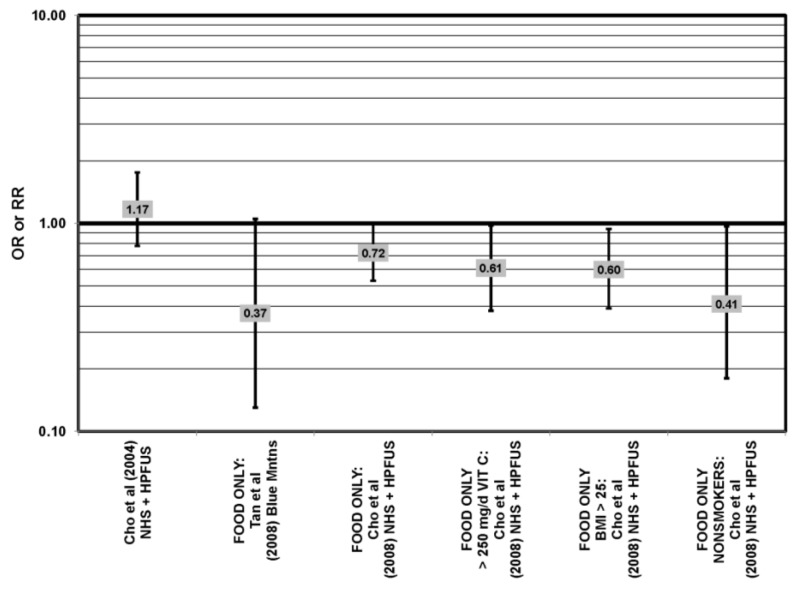
Neovascular AMD odds or risk ratio; high *vs.* low intake of lutein and zeaxanthin: prospective studies.

**Figure 33:**
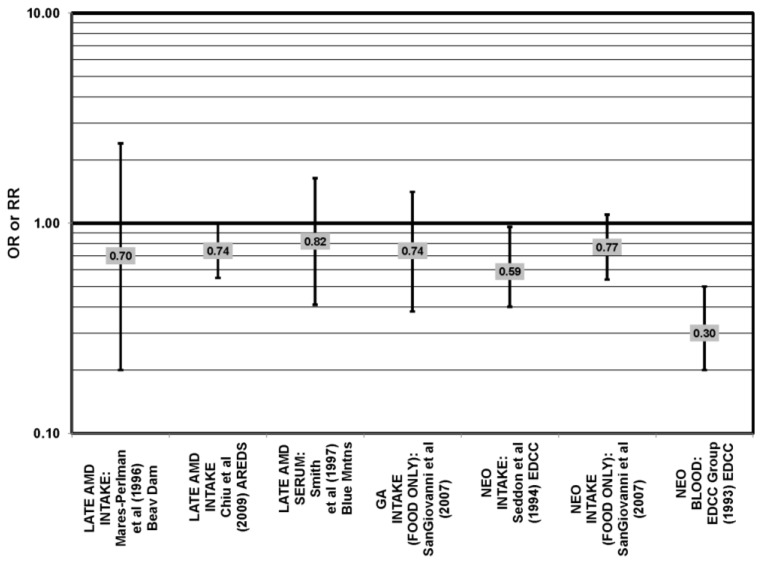
Odds or risk ratio for late AMD, severe AMD, geographic atrophy (GA), exudative AMD (EXUD) or neovascular AMD (NEO); high *vs.* low intake or blood levels of beta-carotene: retrospective and cross-sectional studies.

**Figure 34:**
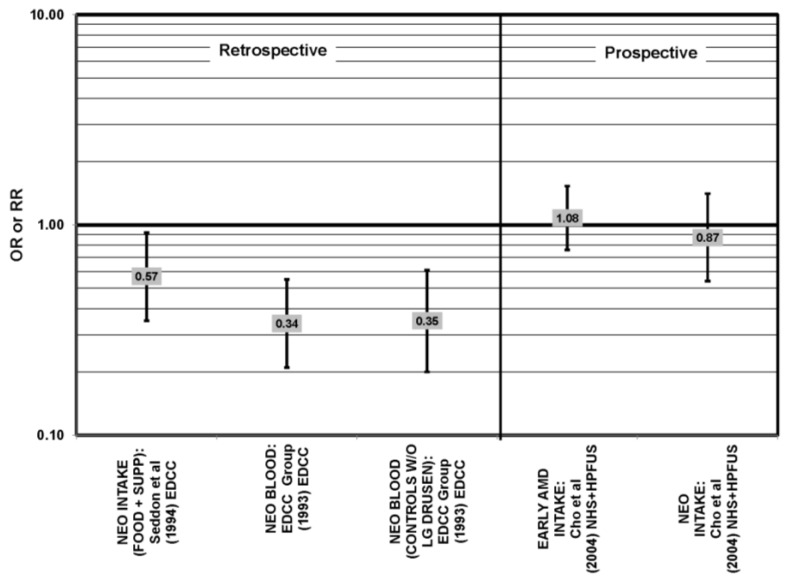
Odds or risk ratio for exudative AMD (EXUD) or neovascular AMD (NEO); high *vs.* low intake or blood levels of total carotenoids: retrospective and cross-sectional studies.

Lutein and zeaxanthin are the only carotenoids found at appreciable levels in the macula [49,62,63,64,65,66] (Figure 24, Figure 25, Figure 26, Figure 27, Figure 28, Figure 29, Figure 30, Figure 31, Figure 32, Figure 33, Figure 34). In addition, their biophysical and biochemical capacities may play a role in the pathogenesis and progression of retinal diseases. For example, lutein and zeaxanthin have the ability to absorb blue light before it reaches the photoreceptors [67,68]. Furthermore, their concentration and potential biologic function may be modified by diet or supplement use.

The EDCC found that high intakes (OR = 0.43; 95% CI: 0.20, 0.70; *p* < 0.001), as well as blood levels (OR = 0.30; 95% CI: 0.20, 0.60; *p* < 0.001) of lutein/zeaxanthin were protective against neovascular AMD [69,70] (Figure 29). Another case control study of 72 patients and 66 controls corroborated this relationship and found that the prevalence of neovascular AMD was reduced in patients with the highest intake of lutein compared to those with the lowest (OR = 0.19; 95% CI: 0.05, 0.67) [71] (Figure 29).

A number of cross-sectional studies support such findings. A study of 380 elderly English men and women found that those with the highest plasma levels of zeaxanthin had less risk for any stage of AMD than those with the lowest plasma levels (OR = 0.50; 95% CI: 0.24, 1.00). The associations between AMD risk and plasma lutein or plasma lutein/zeaxanthin showed the same trend, but were non-significant [72] (Figure 24). Cross-sectional analysis of the POLA (Pathologies Oculaires Liées à l’Age Study) cohort revealed that those with the highest levels of zeaxanthin (OR = 0.07; 95% CI: 0.01, 0.58) or a combination of lutein and zeaxanthin (OR = 0.21; 95% CI: 0.05, 0.79) in their blood had a reduced risk for any stage of AMD, although the amount of lutein alone in the blood (OR = 0.31; 95% CI: 0.09; 1.07) was not significantly associated with disease risk [73] (Figure 24).

Cross-sectional analysis of NHANES III revealed that those aged 40–59 who consumed the highest levels of dietary lutein and zeaxanthin had less risk for pigment abnormalities compared to those who consumed the lowest amounts (OR = 0.10; 95% CI: 0.10, 0.30) (Figure 25). However, these inverse relationships were not observed in the overall study population or among specific ethnic groups for intake or blood levels of lutein and/or zeaxanthin (Figure 25, Figure 26). There was no relationship between lutein/zeaxanthin blood levels and risk for soft drusen among any age group of Non-Hispanic whites in the NHANES III cohort, nor was there a relationship between lutein/zeaxanthin intake and risk for soft drusen among those aged 40–79 (Figure 27). Interestingly, 60–79-year-olds consuming the highest level of lutein/zeaxanthin did have a reduced risk for late AMD (OR = 0.10; 95% CI: 0.00, 0.90) (Figure 28). However, this inverse relationship was not observed in the overall NHANES III population [74]. Data from this cohort suggests that the associations of lutein and zeaxanthin on risk for disease are highly related to the age of those consuming these carotenoids.

Cross-sectional analysis at baseline of 4003 participants in the AREDS study indicated that those in the third quartile of consumption of lutein/zeaxanthin had 20% reduced risk for drusen compared to those in the first quartile of intake (OR = 0.80; 95% CI: 0.67, 0.97). Those in the third quartile of lutein/zeaxanthin intake also had a reduced risk for late AMD (OR = 0.66; 0.49, 0.90) (Figure 28). However, the association became non-significant in those subjects with the highest consumption of lutein/zeaxanthin for both outcomes [52].

The beneficial roles of lutein and zeaxanthin in retina health are supported in data from small intervention studies. Elderly men and women supplemented with 13.7 g/day lacto-wolfberry (a potent source of zeaxanthin) for 90 days increased serum zeaxanthin levels and antioxidant capacity in serum and also reduced pigment changes and soft drusen accumulation relative to the placebo group [75]. A small trial of 108 German men and women supplemented daily with 12 mg lutein and 1 mg zeaxanthin for six months increased MPOD compared to placebo (*p* < 0.001), a change which was maintained even after supplementation stopped [76]. Another placebo controlled trial of 49 elderly women showed that 12 mg/day of lutein supplementation significantly increased MPOD after four months [49]. In another study of 100 men and women, it was shown that increases in MPOD following lutein supplementation were dose-dependent [55,77]. In a small study of 36 subjects, a statistically significant increase in MPOD and improvements in visual acuity and contrast sensitivity were seen in subjects supplemented with 10 mg lutein, 2 mg zeaxanthin and 10 mg meso-zeaxanthin [78]. In a study of 108 early AMD patients, significant increases in MPOD were seen in those supplemented with 20 mg lutein/day and in those supplemented with 10 mg/day lutein plus 10 mg/day zeaxanthin [79]. Data from these trials should be regarded with caution, because of their short duration and limited controls and/or clinical endpoints.

The Zeaxanthin and Visual Function study was a one-year randomized double-masked placebo-controlled study to evaluate the benefits of zeaxanthin, separate from lutein, on visual function, as well as the effects of combining lutein and zeaxanthin in patients with atrophic AMD. Patients received either 8 mg zeaxanthin/day, 9 mg lutein/day (“faux placebo” group) or a combination of both carotenoids. After one year, MPOD increased in all three groups and was not different among the groups. Patients taking zeaxanthin experienced the greatest improvement in high contrast visual acuity and clearance of central scotomas, which are areas of partially diminished or entirely degenerated visual acuity surround by a normal field of vision. Patients taking lutein experienced the most improvement in low contrast acuity and glare recovery. Patients supplemented with both carotenoids saw the least improvement overall, which was attributed to a competition between the carotenoids in the retina [80]. Similarly, a majority of AMD patients given Ocuvite (supplement of 12 g lutein, 1 g zeaxanthin and antioxidants) in the Lutein Nutritional Effects Measured by Autofluorescence (LUNA) study showed elevated MPOD. However, a significant minority did not, perhaps owing to different absorption capacities between individuals.

The Carotenoids in Age-Related Maculopathy in Italians Study (CARMIS) was a randomized intervention in which patients received a combination of 10 mg lutein, 1 mg zeaxanthin, 4 mg astaxanthin and an antioxidant supplement or did not receive any supplements. After 24 months, those receiving supplements had improved and stabilized visual acuity (*p* = 0.003), as well as improved contrast sensitivity at both 12 and 24 months (*p* = 0.001), compared to the non-supplemented group [81].

The Lutein Antioxidant Supplement Trial (LAST) was a randomized, double-masked, placebo-controlled trial of 90 atrophic AMD patients who either received 10 mg lutein, 10 mg lutein with an antioxidant supplement or a maltodextrin placebo. In both groups that received lutein, there was an improvement in MPOD (*p* < 0.05), visual acuity (*p* < 0.05) and contrast sensitivity (at six, 12, 18 degrees, *p* < 0.05) after 12 months of supplementation. The group receiving lutein alone also had improvements in the Amsler grid (*p* < 0.01), a grid of horizontal and vertical lines used to monitor the central visual field and detect visual disturbances, and in glare recovery (AREDS stage II, *p* = 0.02); AREDS stage IV, *p* = 0.05) [82]. In follow-up analysis it was observed that those patients with the greatest increases in MPOD with lutein supplementation were those with the lowest baseline levels, suggesting that lutein supplementation is most beneficial for high risk patients [83]. The data also indicates a saturation of carotenoids in the macula [84].

**Figure 35:**
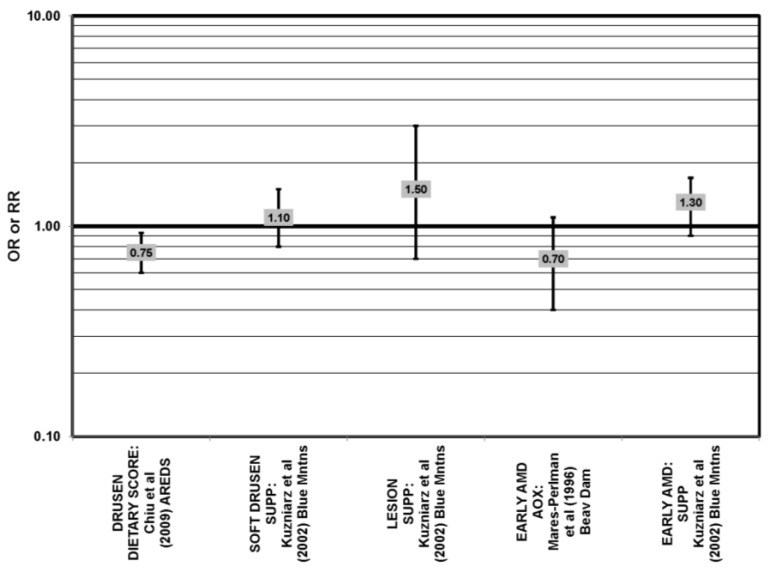
Odds or risk ratio for early AMD indicators (SOFT DRUSEN, LESION) or early AMD; high *vs.* low antioxidant index (AOX) intake or use of multivitamin supplements: retrospective and cross-sectional studies.

Some studies did not find any associations of lutein and zeaxanthin on risk for AMD. Case control studies of AMD patients, including data from the Beaver Dam Eye study and AREDS, did not find any difference in blood levels of lutein/zeaxanthin between patients and controls, nor did they report any effect of plasma lutein/zeaxanthin levels on risk for AMD [85,86,87] (Figure 24, Figure 29). Cross-sectional studies reported similar findings. A study comprised of 722 elderly Japanese reported no difference in serum lutein/zeaxanthin between those with and without late AMD. Analysis of EUREYE and the Beaver Dam Eye study also found no effect of lutein/zeaxanthin intake on risk for early or late AMD [88,89,90] (Figure 28, Figure 29). Prospective studies also did not find an effect of lutein and zeaxanthin intake on AMD risk or incidence of pigment abnormalities. The Rotterdam cohort found no association between any stage of AMD and intake of lutein/zeaxanthin [91]. Data from the Blue Mountains Eye Study found no effect of lutein and zeaxanthin on risk for early AMD five years after baseline (OR = 1.00, 95% CI: 0.60. 1.60), on risk for atrophic or neovascular AMD ten years after baseline (OR = 0.66; 95% CI: 0.48, 0.92) or on risk for drusen 10 years after baseline [92,93] (Figure 34, Figure 35). Similarly, past or present intake of lutein did not affect the five year incidence of pigment abnormalities in the Beaver Dam Eye study [94]. Analysis of men and women in the NHS + HPFUS revealed that those with the highest intakes of lutein and zeaxanthin did not have significantly less risk for early AMD than those with the lowest intakes after adjusting for smoking status, BMI, age, energy intake, alcohol intake, fish intake and use of hormone replacement therapy, and subsequent analyses did not find an association between early or neovascular AMD and lutein/zeaxanthin intake [95,96] (Figure 30, Figure 31, Figure 32). There was slightly lower risk for neovascular AMD among those who consumed high levels of lutein and zeaxanthin from food (RR = 0.72; 95% CI: 0.53, 0.99), and this effect became more robust in subgroups of this population (Figure 35). Men and women who consumed >250 mg/day vitamin C (RR = 0.61; 95% CI: 0.38, 0.98) had a BMI of at least 25 (RR = 0.60; 95% CI: 0.39, 0.94) and were nonsmokers (RR = 0.41; 95% CI: 0.18, 0.97; *p* = 0.07, for trend); the highest level of lutein/zeaxanthin intake was associated with a protective effect on neovascular AMD [96] (Figure 32).

Finally, retrospective analysis of the NHANES III cohort suggested that elevated intake of these carotenoids may increase risk for drusen appearance and AMD, with the effect appearing to be driven by the most elderly participants [74]. In addition, a prospective study of 254 early AMD patients showed that lutein/zeaxanthin intake was associated with increased risk for any stage of AMD after controlling for age, smoking, family history, source study and follow-up duration [55] (Figure 27, Figure 30).

### 5.2. Beta-Carotene

Beta-carotene is another carotenoid that has been investigated in many studies for its potential ability to modulate AMD risk. The EDCC found that increased consumption (OR = 0.59; 95% CI: 0.40, 0.96; *p* = 0.03) and blood levels (OR = 0.30; 95% CI: 0.20, 0.50; *p* < 0.001 for trend) of beta-carotene reduced the risk for neovascular AMD [69,70] (Figure 33). Further support was found in a cross-sectional study of 722 elderly Japanese that reported that those with late AMD had significantly lower serum levels of beta-carotene (*p* < 0.05) [88].

The remainder of studies evaluating the role of beta-carotene did not find an effect of this carotenoid on risk for AMD. Case control studies, including the Blue Mountain Eye Study and Beaver Dam Eye Study, did not find any difference in blood beta-carotene levels between AMD patients and controls and found no association between serum beta-carotene levels and risk for AMD [85,86,97,98]. Data from cross-sectional studies, including data from NHS, Beaver Dam Eye Study and AREDS, are consistent with findings that beta-carotene has no effect on risk for pigment abnormalities, AMD or drusen, respectively [52,90,97,99]. Prospective data from the Rotterdam cohort and Baltimore Longitudinal Study of Aging show no association between beta-carotene intake or serum levels of beta-carotene, respectively, and risk for any stage of AMD. Similarly, in the Blue Mountains Eye study, there was no association between beta-carotene intake and early AMD five years after baseline or late AMD 10 years after baseline [92]. In the NHS + HPFUS, there was also no association between beta-carotene intake and neovascular AMD [95]. The Beaver Dam Eye Study found no association between past or present intake of beta-carotene and risk for pigment abnormalities [94]. The Alpha Tocopherol Beta Carotene Study (ATBC) found that beta-carotene supplementation alone had no effect on AMD risk (OR = 1.04; 95% CI: 0.74, 1.47) [91,92,93,94,95,100,101] (Figure 33).

### 5.3. Alpha-Carotene

In addition to beta-carotene, the EDCC also evaluated the effect of the isomer, alpha-carotene, on risk for AMD. In this study, higher blood levels of alpha-carotene (OR = 0.50; 95% CI: 0.30, 0.80; *p* = 0.003, for trend) were associated with decreased risk for neovascular AMD [70]. Prospective analysis of the Beaver Dam Eye Study also found that those with an elevated past intake of dietary alpha-carotene were at a reduced risk for the appearance of large drusen (OR = 0.52; 95% CI: 0.30, 1.00) [94]).

The remainder of case control and prospective studies, as well as more recent NHS data reported that alpha-carotene does not significantly affect risk for AMD. Case-control analysis of patients in the Beaver Dam Eye Study, EDCC and three additional studies did not find any association between serum levels of alpha-carotene and any stage of AMD or any difference in blood levels of alpha-carotene between AMD patients and controls [69,85,86,90,98,102].

### 5.4. Lycopene

Lycopene is commonly found in the American diet in tomato-based products [103]. Evidence for a potential role of lycopene in retina health was found in a small case control study, which found that AMD patients had less lycopene in their serum, LDL and HDL than in healthy controls (*p* = 0.006) [85]. Similarly, a nested case-control study of 167 patients from the Beaver Dam Eye Study found that higher lycopene blood levels were associated with decreased risk for any stage of AMD (OR = 0.45; 95% CI: 0.22, 0.91) [98].

The remaining observational, cross-sectional and prospective studies did not find a relationship between lycopene and AMD. The EDCC found no association between intake or blood levels and risk for neovascular AMD (OR = 0.80; 95% CI: 0.50, 1.30; *p* = 0.4, for trend) [69,70]. Furthermore, cross-sectional analysis of elderly Japanese found no association between serum lycopene levels and late AMD risk, and cross-sectional analysis of the Beaver Dam Eye Study found no effect of lycopene intake and risk for early or late AMD. Prospective studies, such as the Rotterdam cohort and Blue Mountains Eye study, found no association between lycopene intake and any stage AMD or early AMD, respectively (HR = 1.01; 95% CI: 0.97, 1.04) (OR = 0.80; 95% CI: 0.50, 1.40) [91,92]. Recent NHS data found no association between serum lycopene levels and risk for pigment abnormalities, and the NHS + HPFUS found no association between early or neovascular AMD and lycopene intake [95].

### 5.5. Cryptoxanthin

Cryptoxanthin is a carotenoid found in foods, such as avocado, basil and mango [103]. The EDCC and a cross-sectional analysis of 722 elderly Japanese found that increased blood levels of cryptoxanthin were associated with decreased risk for any stage of AMD [88]. The remaining studies did not find any associations between cryptoxanthin and AMD. A case control study did not find any difference in blood cryptoxanthin levels between patients and controls [85]. The Beaver Dam Eye study found no association between serum levels of beta-cryptoxanthin and risk for any stage of AMD [98]. The EDCC Beaver Dam Eye study, Rotterdam study, Blue Mountains Eye study and NHS + HPFUS also showed that intake of cryptoxanthin had no effect on risk for neovascular, early or late AMD [69,90,91,92,95].

### 5.6. Total Carotenoids

Total carotenoid levels have also been analyzed for their potential to confer retinal benefit. In the EDCC, increasing intake of total carotenoids was associated with decreased risk for neovascular AMD (OR = 0.57; 95% CI: 0.35, 0.92; *p* = 0.02, for trend) [69], and analysis of blood levels corroborated these relationships: OR = 0.34 (95% CI: 0.21, 0.55; *p* < 0.001, for trend) (Figure 34) [70]. Similar results were obtained when AMD cases were compared to controls without large drusen. Another smaller case-control study with 48 AMD patients found that total carotenoid plasma levels were significantly lower in patients with late AMD compared to those in the early stages of the disease (*p* < 0.05), although there was no difference in plasma levels between AMD patients and healthy controls [104]. This may imply that carotenoids play a more important role in disease progression rather than disease onset. This finding is comparable to that of another case-control study of 197 Japanese men and women that reported that eyes with early AMD and the normal eye of an AMD patient had comparable macular carotenoid levels, which were both higher than those of an eye with late AMD (*p* < 0.001). However, in this study, macular carotenoid levels of all AMD patients were also significantly lower than those of age-matched healthy subjects (*p* < 0.001), as determined by Raman spectroscopy [105].

Despite these reported beneficial effects of carotenoids on the retina, four case-control studies did not find any difference in blood levels of total carotenoids between AMD patients and healthy controls [85,86,106,107]. Furthermore, prospective analyses of the NHS + HPFUS found no association between early or neovascular AMD and total carotenoid intake [95] (Figure 34).

### 5.7. Summary

Taken together, these observational studies suggest that of all of the carotenoids studied, lutein and zeaxanthin may confer the most benefit to the retina. These benefits may be specific for certain types or stages of AMD. Advanced AMD shows the most consistent inverse relationships between lutein intake and risk for lesions. Data concerning the effects of lutein and zeaxanthin on outcomes, such as visual acuity and contrast sensitivity, suggest that 10 mg of lutein/day, without zeaxanthin, may confer the most retinal benefit. That said, intervention studies, such as AREDS II, will clarify the role of these carotenoids in the risk for onset and progression of specific AMD outcomes.

## 6. Vitamin A

Analysis of vitamin A/retinol intake and blood levels has also been examined for its role in retinal health, as many carotenoids are byproducts of vitamin A. NHANES I found that those who consumed increased amounts of fruits and vegetables rich in vitamin A had a decreased risk for any stage of AMD (OR = 0.59; 95% CI: 0.37, 0.99) [108]. Also, a prospective analysis of the Beaver Dam Eye Study found that those with an elevated past intake (OR = 0.53; 95% CI: 0.30, 1.00) or current intake (OR = 0.45; 95% CI: 0.20, 1.00) of pro-vitamin A carotenoids were at a reduced risk for the appearance of large drusen [94].

Case control analyses found that patients and healthy controls did not have different plasma levels of vitamin A and that plasma levels were not different in different stages of AMD [86,104,106,107]. Additionally, EDCC and AREDS found that intake of retinol did not modulate risk for neovascular AMD [69,99]. Cross-sectional analyses found no relationship between intake of vitamin A rich food and AMD risk in NHANES III or between intake of retinol or vitamin A and pigment abnormalities in the NHS [102,108]. Prospective analysis of the Blue Mountain Eye study found that vitamin A and retinol intake did not affect risk for AMD [92]. Additionally, prospective data from the Baltimore Longitudinal study showed no effect of plasma retinol levels and severe AMD risk, and the NHS + HPFUS found no association between early or neovascular AMD and vitamin A intake [95,100]. Analysis of four examinations from the Beaver Dam Eye Study showed that supplementation with vitamin A was associated with an increased risk for late AMD (OR = 3.05; 95% CI: 1.60, 5.82) [109]. In summary, the epidemiologic data regarding the relationship between vitamin A/retinol intake and blood levels and the risk of AMD are mixed, and no reliable trend can be reported.

## 7. Vitamin E

Vitamin E (alpha-tocopherol) is a lipophilic antioxidant that some studies suggest has a role in retina health and diminishing risk for AMD. A case-control study of 48 AMD patients indicated that plasma vitamin E levels were significantly lower in patients with late AMD compared to patients with early AMD or healthy age-matched controls (*p* < 0.05), and these effects remained even after adjusting for cholesterol levels. This adjustment is of interest, because vitamin E is lipid soluble and associates with cholesterol on lipoprotein particles [104]. Another case-control study of 25 elderly AMD patients found that vitamin E serum levels were significantly lower in AMD patients compared to healthy controls (*p* < 0.001). In this study, patients and controls had similar cholesterol levels [110]. A third case-control study found that comparison of serum vitamin E levels in 35 AMD patients with 66 controls showed that vitamin E was inversely associated with risk for AMD [107].

Cross-sectional analysis of the 2584 participants of the POLA study showed that after adjusting for plasma lipid levels, those with the highest levels of plasma vitamin E had a reduced risk for signs of early AMD, such as pigment changes or soft drusen (OR = 0.72; 95% CI: 0.53, 0.98; *p* = 0.04, for trend) and late AMD (OR = 0.18; 95% CI: 0.05, 0.67; *p* = 0.004, for trend) [111]. These associations remained even in populations of different ethnicity. A cross-sectional study of 722 elderly Japanese found that patients with late AMD had marginally significant lower levels of serum alpha-tocopherol than healthy controls (*p* = 0.056) [88]. Baseline analysis of 4003 participants of the AREDS cohort indicated that those consuming the highest amounts of vitamin E had a marginally reduced risk for late AMD compared to those consuming the smallest amounts (OR = 0.66; 95% CI: 0.45, 0.99; *p* = 0.052, for trend) [52].

Some prospective study data corroborate the cross-sectional data. The Baltimore Longitudinal Study of Aging indicated that among 976 men and women, those with the highest plasma tocopherol levels were at a reduced risk for any stage of AMD, even after adjusting for age, gender and nuclear opacity (OR = 0.43; 95% CI: 0.25, 0.73) [100]. In the Rotterdam prospective cohort, vitamin E intake was also associated with a slight reduction in risk for AMD (HR = 0.92; 95% CI: 0.84, 1.00) [91]. Among 498 women in the NHS, increasing amounts of vitamin E intake were associated with a decreased prevalence of pigment abnormalities, an indicator of early AMD [102].

There is also a significant amount of case control, prospective and intervention data suggesting that there is no relationship between vitamin E levels and AMD risk. A case-control study of 167 cataract patients from the Beaver Dam Eye Study revealed that there was no association between alpha-tocopherol (OR = 0.80; 95% CI: 0.40, 1.50) serum levels or gamma-tocopherol (OR = 1.30; 95% CI: 0.70, 2.40) serum levels and any stage of AMD. The EDCC and an additional study of 26 neovascular patients also found no association between serum vitamin E levels and risk for AMD [70,98,106]. Furthermore, a case-control study with 34 AMD patients found no difference in serum, LDL or HDL levels of alpha- or gamma-tocopherol between AMD patients and healthy controls [85]. Additionally, case-control studies comprised of 56–165 AMD patients found no difference in blood alpha-tocopherol levels between patients and healthy controls [86,97,112]. The EDCC found no association between vitamin E intake (OR = 1.07; 95% CI: 0.63, 1.84; *p* = 0.98, for trend) or supplementation (OR = 0.97; 95% CI: 0.6, 1.50, *p* = 0.28, for trend) and risk for neovascular AMD (OR = 1.46; 95% CI: 0.88, 2.44) [69]. Similarly, in case-control analysis of the AREDS, there was no association between vitamin E intake and risk for drusen, geographic atrophy or neovascular AMD [99].

Cross-sectional analysis of the AREDS population revealed that intake of vitamin E was not associated with risk for drusen [52]. Retrospective analysis of the Beaver Dam cohort (*n* = 1968) also indicated that past intake of vitamin E with or without supplementation did not have a significant effect on early or late AMD [90]. Cross-sectional analysis of 4753 elderly men and women found no increased risk for neovascular AMD in those with the lowest plasma levels of alpha-tocopherol (OR = 0.82; 95% CI: 0.47, 1.41) [89]. In prospective analysis of the Beaver Dam cohort, past intake of vitamin E was associated with a decreased risk for large drusen five years after baseline (OR = 0.40; 95% CI: 0.20, 0.90; *p* = 0.04 for trend), but there was no association with present vitamin E intake or past or present vitamin E supplementation, nor was there an association between past or present intake of vitamin E and risk for pigment abnormalities [94]. In the Physicians’ Health Study, vitamin E supplementation was not associated with risk for any stage of AMD (RR = 0.87; 95% CI: 0.53, 1.43) [113]. Similarly, a prospective analysis of 118,428 men and women who participated in the NHS + HPFUS showed that vitamin E intake had no effect on risk for early or neovascular AMD [95].

The Women’s Health Study (WHS), a randomized double-masked placebo controlled trial, gave 39,421 women either 600 IU vitamin E every other day or placebo for 10 years. After adjusting for age, aspirin intake and beta-carotene, vitamin E had no effect on risk for visually significant AMD (RR = 0.93; 95% CI: 0.72, 1.19), late AMD (RR = 1.13; 95% CI: 0.67, 1.92) or AMD with or without vision loss (RR = 0.90; 95% CI: 0.77, 1.06) [114]. The ATBC Study gave subjects either daily supplements of 50 mg vitamin E, 20 mg beta-carotene, both or placebo, and after five to eight years of intervention, alpha-tocopherol supplementation alone had no effect on risk for AMD (OR = 1.13; 95% CI: 0.81, 1.59) [101]. The Vitamin E and Age-related Cataract and Maculopathy (VECAT), a clinical trial of 1193 elderly participants, indicated that supplementation with 500 IU vitamin E for four years also had no effect on incidence of early AMD (RR = 1.05; 95% CI: 0.69, 1.61) or late AMD (RR = 1.36; 95% CI: 0.67, 2.77) [115]. Lastly, in a randomized, double masked, placebo-controlled trial of 14,236 male physicians over the age of 50, alternate day use of 400 IU of vitamin E for an average of eight years had no effect on diagnosis of AMD (HR, 1.03; 95% CI, 0.78–1.37) [116].

A 10 year follow-up of subjects from the Blue Mountains Eye study showed those with the highest intakes of vitamin E were at greater risk for late stage atrophic AMD (RR = 2.55; 95% CI: 1.14, 5.70), and there was no association between vitamin E intake and neovascular AMD (RR = 1.96; 95% CI: 0.74, 5.15) [93]. Analysis of all four examinations of the Beaver Dam Eye study reported a positive association between vitamin E supplementation and incident late AMD (OR = 1.78; 95% CI: 1.04, 3.05) [109].

While there is a large body of evidence to say there is an inverse relationship between vitamin E consumption and risk for AMD, there is also a substantial amount of evidence to say that no relationship exists. Some evidence even suggests a direct relationship with certain stages of AMD. Overall, there is not a consistent relationship from the data to date.

## 8. Vitamin C

Case control, observational and prospective analyses do not suggest that vitamin C plays a large role in modulating AMD risk. This includes exposure to vitamin C in blood supplements or diet, some of which monitored intake over 10 years and/or were from large cohorts [61,62,92,93,94,95,96,98,101,111,112,113,116]. Nevertheless, in a case control study of 48 Italian AMD patients, it was found that patients with late AMD had significantly lower plasma vitamin C levels than those patients with early AMD (*p* < 0.05), but there was no difference in plasma levels of vitamin C between AMD patients and healthy controls [104]. Additionally, a case control study of 56 AMD patients found significantly decreased serum vitamin C levels in AMD patients compared to controls [112]. This may be attributable to the small sample size of the study [89,111,112].

Two different cross-sectional analyses of the baseline data from AREDS (of 4519 and 4403 participants) studied the effects of a single nutrient or groups of nutrients on AMD risk, and both analyses indicated that after multivariate adjustment, vitamin C intake alone had no association with drusen formation, late AMD, geographic atrophy or neovascular AMD [52,99]. Analyses of the Beaver Dam Eye Study found that past intake of vitamin C (with or without supplements) did not have a significant effect on risk for early or late AMD [90]. Prospective analyses, including the Physician’s Health Study, Rotterdam study, Baltimore Longitudinal, Blue Mountains Eye Study, Beaver Dam Eye Study and NHS + HPFUS, also indicate that vitamin C intake or blood levels are not associated with risk for AMD; respectively, [91,93,94,100,113]. In a randomized, double masked placebo controlled trial of 14,236 male physicians, daily supplementation of 500 mg vitamin C for eight years had no effect on risk for AMD (HR, 0.99; 95% CI, 0.75–1.31) [116].

Data collected at four different examinations of the Beaver Dam Eye Study found that use of vitamin C supplements was associated with increased risk for late AMD (OR = 2.47; 95% CI: 1.40, 4.80) [109]. This corroborated data from the Blue Mountains Eye Study, which found that those with the highest intakes of vitamin C had an increased risk for early AMD after five years, compared to those with the lowest intakes of vitamin C (OR = 2.30; 95% CI: 1.30, 4.00; *p* = 0.002, for trend) [92].

Overall, the body of epidemiological evidence does not inform a consistent relationship between Vitamin C and risk for AMD, and further investigation is warranted.

## 9. Antioxidant Combinations or Multivitamins

**Figure 36:**
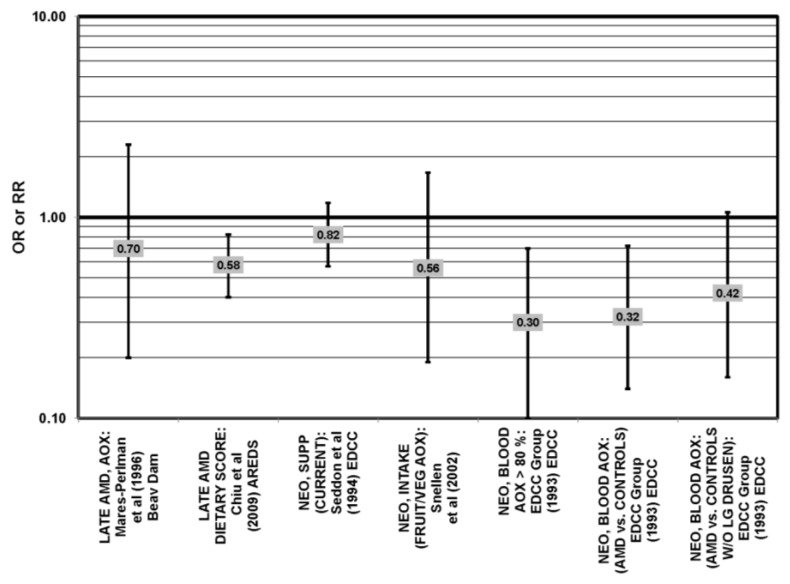
Odds or risk ratio for late AMD, exudative AMD (EXUD) or neovascular AMD (NEO); high *vs.* low antioxidant index (AOX) intake or blood levels or use of multivitamin supplements: retrospective and cross-sectional studies.

The case-control EDCC found no effect of multivitamin supplementation on risk for neovascular AMD (OR = 0.82; 95% CI 0.57, 1.18). There was, however, an association between antioxidant index and late AMD [69]. Participants who had the highest blood levels of selenium, vitamin C or vitamin E, had a reduced risk for neovascular AMD (OR = 0.30; 95% CI: 0.10, 0.70; *p* = 0.005, for trend) compared to those who had the lowest amounts of at least three of the nutrients [70] (Figure 36).

**Figure 37:**
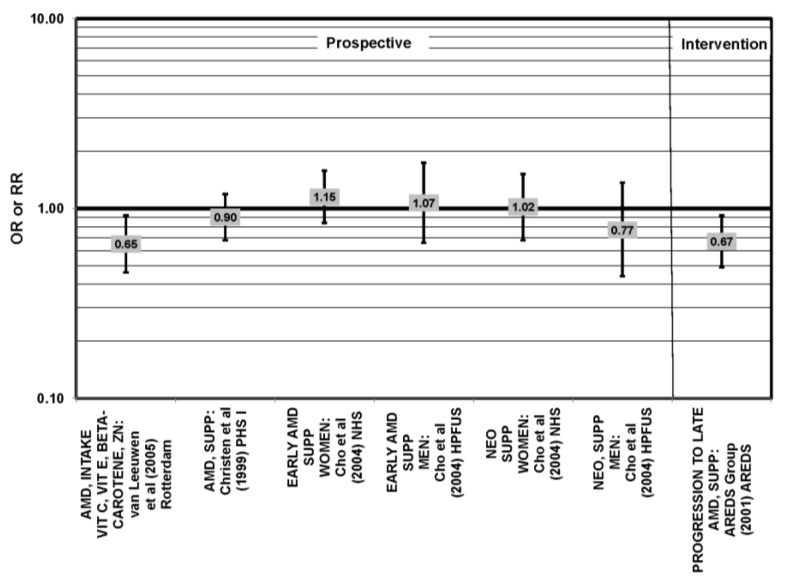
Odds or risk ratio for AMD, early AMD or neovascular (NEO) AMD; high *vs.* low intake of antioxidant combination or use of multivitamin supplement: prospective studies, intervention.

In cross-sectional analysis of 4003 participants in AREDS, a compound score, which included dietary intake of vitamin E, vitamin C, zinc, lutein/zeaxanthin, DHA, EPA and low dietary glycemic index was created to compare overall diet to the risk for AMD. Low scores indicated lowest intake of such nutrients and *vice versa*. Compared to those subjects with the lowest score, those with the highest score were at a reduced risk for drusen (OR = 0.75; 95% CI: 0.60, 0.93; *p* = 0.048, for trend) and late stage AMD (OR = 0.58; 95% CI: 0.40, 0.82; *p* = 0.002, for trend) [52] (Figure 35, Figure 36). Additionally, in prospective analyses, the Rotterdam study indicated that after adjusting for age, sex, BMI, smoking, blood pressure, atherosclerotic score, alcohol intake and total cholesterol, subjects that had intakes of beta-carotene, vitamin C, vitamin E and zinc above the median had reduced risk for AMD (HR = 0.65; 95% CI: 0.46, 0.92) [91] (Figure 37).

In the randomized, double masked, placebo controlled AREDS study, participants either received an antioxidant cocktail or placebo, Results indicated that supplementation with a cocktail of beta-carotene, vitamin E, vitamin C, zinc and copper reduced progression from intermediate to advanced AMD by 34% (95% CI: 0.47, 0.91) over about six years of follow-up [92,117,118] (Figure 37). Another clinical trial of 27 patients with early AMD showed that daily supplementation with 180 mg vitamin C, 30 mg vitamin E, 22.5 mg zinc, 1 mg copper, 10 mg lutein, 1 mg zeaxanthin and 4 mg astaxanthin improved function in the central retina as measured by electroretinogram (*p* < 0.01) (data not shown) [119].

In a double-masked, placebo controlled trial of dry AMD patients, it was shown that supplementation with vitamin E, zinc, magnesium, vitamin B6 and folate for 18 months maintained visual acuity, compared to placebo treatment, in which there was a decrease in visual acuity (*p* = 0.03). The antioxidant supplement group also reported greater vision stability in the areas of visual acuity and contrast sensitivity (*p* = 0.05) [120]. Another double-masked, placebo controlled trial of dry AMD patients found that supplementation with antioxidants and omega-3 fatty acids maintained visual acuity over six months, while the placebo group lost visual acuity (*p* < 0.05) [121].

The Carotenoids with Co-antioxidants in Age Related Maculopathy (CARMA) trial, a randomized, double masked placebo controlled clinical trial, gave 433 adults a daily dose of 12 mg lutein, 12 mg alpha tocopherol, 150 mg vitamin C, 20 mg zinc oxide and 0.4 mg copper gluconate. It was found that eyes in the supplemented group progressed along the AMD severity scale at a slower rate compared to the placebo group. However, there was no statistically significant difference between development of AMD in the supplemented *versus* placebo group [122]. Among the evidence that challenges a role for antioxidants and AMD is a case-control study of 72 cases and 66 controls, which could not find a significant association between antioxidant intake and AMD (OR = 0.56; 95% CI: 0.19, 1.67) [71] (Figure 36). A cross-sectional analysis of 1968 participants of the Beaver Dam Eye Study showed that dietary antioxidants (measured with an antioxidant index) had no association with risk for early or late AMD [90] (Figure 35, Figure 36). A cross-sectional analysis of the baseline data from 2873 participants of the Blue Mountains Eye Study found vitamin supplements to be without effect on risk for early AMD (OR = 1.30; 95% CI: 0.90, 1.70) [123] (Figure 35). There was also no effect on risk for soft drusen (OR = 1.10; 95% CI: 0.80, 1.50) or AMD lesions (OR = 1.50; 95% CI: 0.70, 3.00) [123] (Figure 35). Further, prospective analysis of four examinations of the Beaver Dam Eye Study indicated that multivitamin supplementation had no effect on early or late AMD [109]. Among women in the NHS, as well as men in the HPFUS, there was no association between risk for early or neovascular AMD and use of a multivitamin supplement [95] (Figure 37). Finally, the Physicans Health Study reported that supplementation with a multivitamin had no effect on AMD (RR = 0.90; 95% CI: 0.68, 1.19) [113] (Figure 37).

Epidemiologic support for the value of antioxidant combinations deserves additional study. The AREDS trial indicates that multivitamin supplementing may be beneficial, and several smaller studies have corroborated such results. In addition, some studies have found that a dietary intake high in nutrients with antioxidant properties reduces risk of early AMD in individuals with high genetic risk [124]. Clearly, data from the AREDS II trial should be invaluable to determine whether or not supplementation with individual or specific combinations of nutrients provides advantage with regard to preserving retinal integrity. Furthermore, since multivitamins do not appear to be harmful to the retina, use of a supplement or eating a diet rich in fruits and vegetables may be an advisable practice, as well as consuming a diet rich in antioxidants, especially in those at high genetic risk [25,124].

## 10. Zinc

Zinc is essential for many physiological processes, including immunity, reproduction and neuronal development [125]. Since concentrations of zinc are very high in the retina, it has been hypothesized that zinc supplementation may aid retinal health.

**Figure 38:**
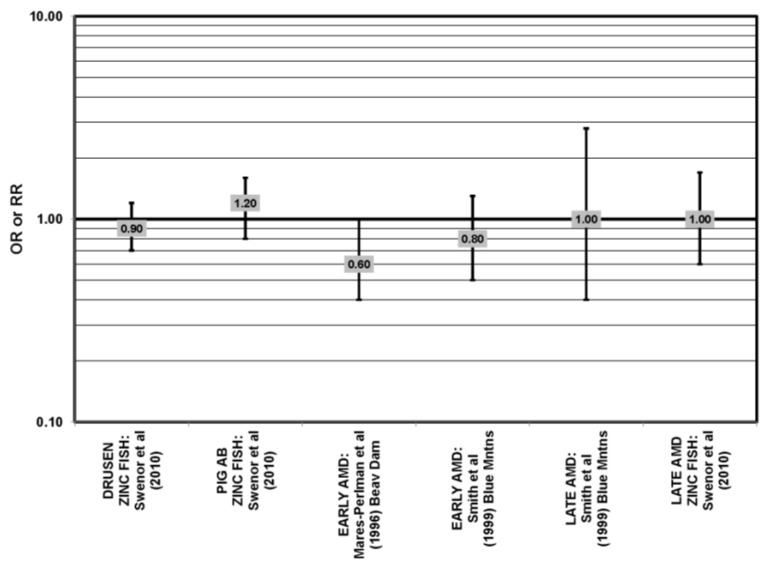
Odds or risk ratio for early AMD indicators, early AMD or late AMD; high *vs.* low intake of zinc: retrospective and cross-sectional studies.

There have been no case-control investigations of zinc and AMD risk. Retrospective analysis of 1968 participants of the Beaver Dam Eye study found that compared to people with the lowest amount of zinc intake from foods, those with the highest amount had a reduced risk for early AMD (OR = 0.60; 95% CI: 0.40, 1.00; *p* < 0.05). There was no association with late AMD [90] (Figure 38).

A cross-sectional study of 44 subjects indicated that while there was no difference in zinc and copper levels in the neural retina (analyzed *ex vivo*) between healthy subjects and those with AMD (*p* > 0.09), there was significantly less zinc and copper in the RPE and choroid in AMD patients compared to healthy subjects (*p* = 0.002) [126].

**Figure 39:**
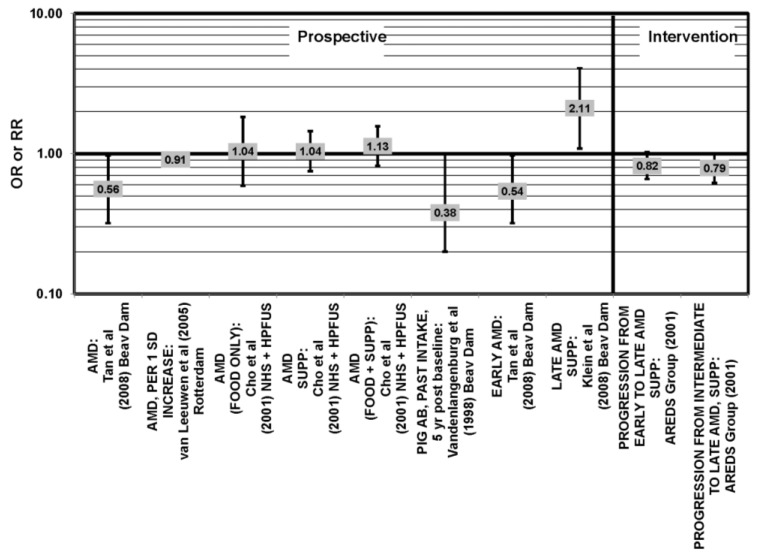
Odds or risk ratio for AMD, early AMD or late AMD; high *vs.* low intake (with or without supplements) of zinc: prospective and intervention studies.

Prospective analysis of the Rotterdam study showed that among 4170 participants, increased consumption of zinc was associated with a reduced risk for any stage of AMD (HR = 0.91; 95% CI: 0.83, 0.98) [91] (Figure 39). Data from 1709 participants of the Beaver Dam Eye Study indicated that there was a decrease in risk for pigment abnormalities five years after baseline for those with past zinc intake (food and supplements) in the highest quintile (OR = 0.38; 95% CI: 0.20, 1.00; *p* = 0.03, for trend) [94] (Figure 39). A 10 year follow-up analysis showed that those with the highest zinc intake were at a reduced risk for any type of AMD (OR = 0.56; 95% CI: 0.32, 0.97), as well as early AMD (OR = 0.54; 95% CI: 0.32, 0.97), compared to all other participants [93] (Figure 39).

In AREDS, participants who consumed zinc were less likely to progress from intermediate to more advanced AMD than those who did not (OR = 0.79; 95% CI: 0.62, 0.99). However, there was no significant effect of zinc supplementation on progression to late AMD when the analysis included those with grade 2 AMD [117] (Figure 39). Other than the AREDS study, there have been a few clinical trials that have analyzed the effect of zinc on AMD. In a study of 90 subjects, Newsome *et al.* found that 100 mg zinc twice a day for two years reduced vision loss (one-tailed *p* = 0.001) [127]. Although this dose of zinc has been thought to induce toxicity in some patients, adverse effects related to zinc toxicity were not observed in the study population [127,128,129]. Another trial of 80 dry AMD patients found that supplementation with 25 mg zinc monocysteine twice a day for six months improved several indicators of retinal function, such as visual acuity, contrast sensitivity and macular flash recovery time (*p* < 0.0001) [130].

A large cross-sectional study of 2,873 participants of the Blue Mountains Eye Study found that zinc had no effect on early AMD [123]. This finding was confirmed in second analysis of slightly more subjects (3654) from the same study for early (OR = 0.80; 95% CI: 0.50, 1.30) or late AMD (OR = 1.00; 95% CI: 0.40, 2.80) [131] (Figure 38). Similarly, five years after baseline, zinc intake had no effect on early AMD incidence [92]. Swenor *et al.* found that compared to those who consumed less than 0.07 servings per week of fish with a high zinc content (for example, crab and oysters), those who consumed more than 0.07 servings per week did not have any change in risk for the appearance of drusen, pigment abnormalities or late AMD [59] (Figure 38).

A prospective study of 104,208 elderly men and women also showed that there was no association between risk for AMD and ten years of zinc intake (RR = 1.13; 95% CI: 0.82, 1.57) [132] (Figure 39). Compared to those with the lowest amount of zinc intake from food, those with the highest food intake did not have a reduced risk of AMD (RR = 1.04; 95% CI: 0.59, 1.83) [132] (Figure 39). There was also no association between AMD risk and zinc supplementation (RR = 1.04; 95% CI: 0.75, 1.45) [132] (Figure 39).

Although two trials have shown that zinc alone can improve vision, a placebo-controlled trial of 112 AMD subjects found that 200 mg zinc per day for two years had no effect on vision [133]. Analysis of the Beaver Dam Eye Study indicated that use of zinc supplements was associated with an increased risk for late AMD (OR = 2.11; 95% CI: 1.09, 4.07) [109] (Figure 39). In summary, the data to date is not robust, and we should await results from AREDS II to determine if zinc supplementation confers protection with regard to retinal function.

## 11. Vitamin D

**Figure 40:**
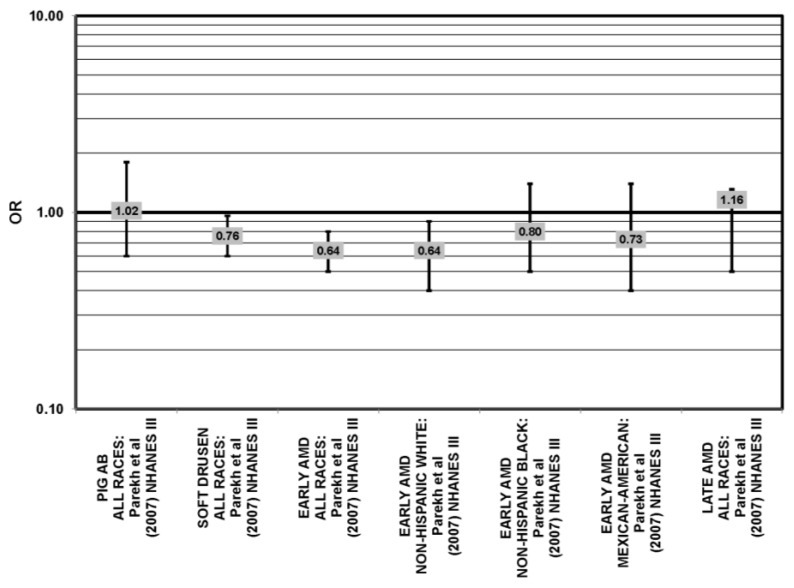
Odds ratio for early AMD, indicators of early AMD (PIG AB, SOFT DRUSEN) or advanced AMD; high *vs.* low serum levels of vitamin D: retrospective studies.

**Figure 41:**
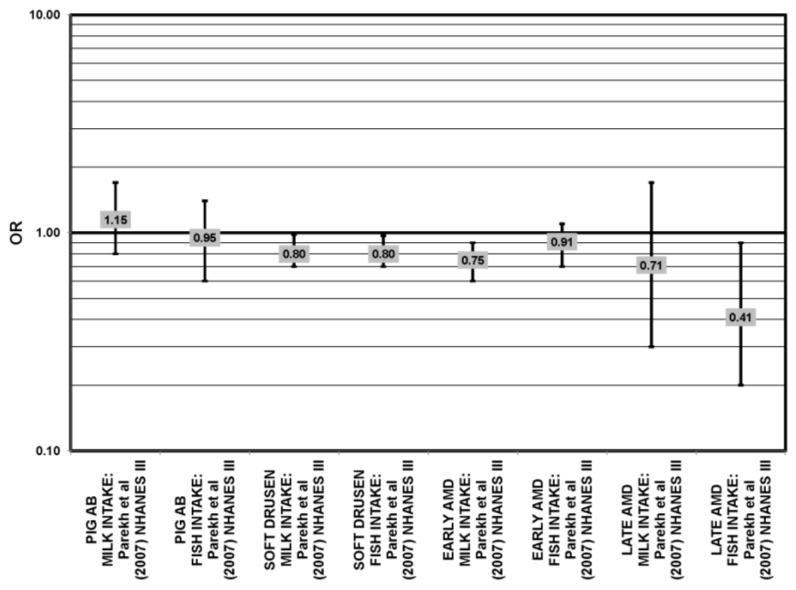
Odds ratio for early AMD, indicators of early AMD (PIG AB, SOFT DRUSEN) or advanced AMD; high *vs.* low intake of milk or fish in those at least 40 years of age: retrospective studies.

**Figure 42:**
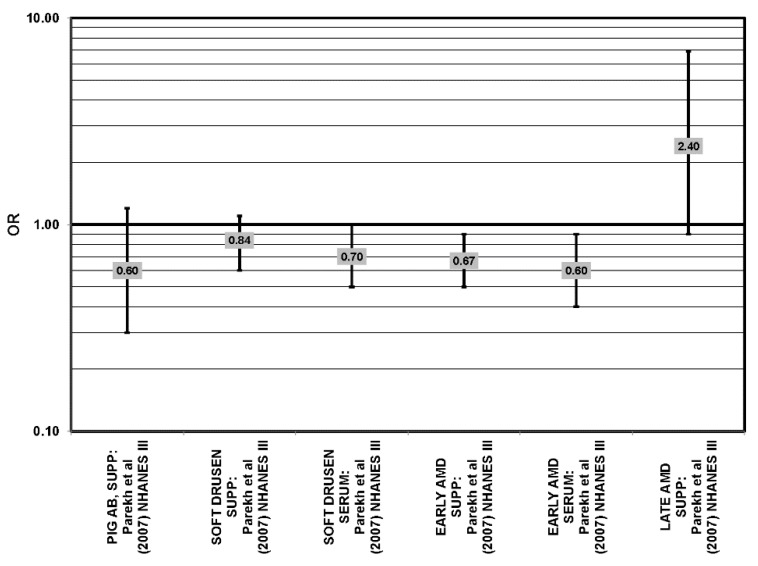
Odds ratio for early AMD, indicators of early AMD (SOFT DRUSEN, PIG AB) or advanced AMD upon vitamin D supplementation in in those at least 40 years of age who do not consume milk daily: retrospective studies.

There have been only a few observational studies regarding the role of vitamin D in ameliorating AMD risk [134] (Figure 40, Figure 41, Figure 42). Parekh *et al*. found that among 7752 participants of NHANES III, non-Hispanic whites with the highest serum levels of 1,25(OH)_2_ vitamin D had a reduced risk for early AMD (OR = 0.64; 95% CI: 0.40, 0.90) compared to those with the lowest serum levels (Figure 40). However, there was no beneficial effect in non-Hispanic blacks or Mexican Americans (Figure 40). Combining all races, there was also a reduced risk for soft drusen in those with the highest serum levels of 1,25(OH)_2_ vitamin D compared to those with the lowest levels (OR = 0.76; 95% CI: 0.60, 0.96) (Figure 40). There was no effect of serum levels of vitamin D on the appearance of pigment abnormalities or advanced AMD (Figure 40). Adjustments for sex and other covariates did not influence the observations [134].

Millen *et al.* examined 1313 postmenopausal women and found a protective association of vitamin D status with the prevalence of AMD. In women younger than 75 years who had 25-hydroxyvitamin D (25[OH]D) concentrations higher than 38 nmol/L, there was a 48% decrease in the odds of early AMD. Adjustment for BMI and physical activity attenuated this observation. However, a statistically significant direct association between 25(OH) D and early AMD was observed in women aged 75 years or older (*p* for trend = 0.05). Additionally, significantly decreased odds of developing early AMD were observed among women in the fifth quintile for vitamin D intake [135].

Milk and fish are two of the most common sources of vitamin D in the American diet, and in exploratory analyses of the NHANES III study, it was found that among participants at least 40 years of age, those who consumed milk at least daily were at a reduced risk for early AMD (OR = 0.75; 95% CI: 0.60, 0.90) and soft drusen (OR = 0.80; 95% CI: 0.70, 0.98), compared to those who consumed it less than weekly (Figure 41). Pigment abnormalities and advanced AMD were not affected by milk consumption (Figure 41). In CAREDS, among women under age 75, those with the highest consumption of low-fat dairy products were at a reduced risk for intermediate AMD (OR = 0.50; 95% CI: 0.30, 0.80) [54] (Figure 15). Those over the age of 40 who consumed fish at least once a week were at a reduced risk for soft drusen (OR = 0.80; 95% CI: 0.70, 0.97) and advanced AMD (OR = 0.41; 95% CI: 0.20, 0.90), compared to those who consumed fish less than twice a month (Figure 41). For those in this age group that did not consume milk daily, supplementation did attenuate early AMD risk (OR = 0.67; 95% CI: 0.50, 0.90), but not risk for soft drusen, pigment abnormalities, or advanced AMD [134] (Figure 42). Although risk for early AMD did not correspond to risks associated with early AMD indicators, results of this study do leave open the possibility that vitamin D may help decrease risk for certain forms of AMD. The majority of data suggest a protective effect of Vitamin D on risk of certain forms of AMD.

## 12. Dietary Patterns

Diet is a potentially modifiable risk factor for AMD. As shown in the above sections, different nutritional factors may influence the development and/or progression of AMD. However, dietary components may interact with each other, either in a single food or a meal, and alteration in intake or elimination of one food may increase intake of another [136]. As shown above, associations of single nutrients and AMD are often inconsistent across studies. In addition, it is often difficult to disentangle single aspects of a diet or lifestyle from each other [137]. Examining overall diet quality in relation to AMD may better account for the relationships among different diet components [136].

A case control study of 696 men and women (437 AMD patients and 259 unrelated controls), measured dietary information using a 97-item block food frequency questionnaire, Health Habits and History Questionnaire (HHHQ)-DietSys Analysis software, Healthy Eating Index (HEI) and the Alternate Healthy Eating Index (AHEI). Those who were in the highest quartile, compared to the lowest, of diet quality using the AHEI, but not HEI, score were at significantly decreased odds of AMD (0.54, 95% CI 0.30–0.90). This may be due to the fact that the AHEI utilizes specific modifications to incorporate nutritional parameters related to chronic disease, such as emphasis on fat quality in addition to quantity [136]. In a study of 2005 women enrolled in CAREDS, diet quality was measured using a modified 2005 Healthy Eating Index score, and a six point healthy lifestyle score (HLS). Women in the highest quintile compared to the lowest for the mHEI score had 46% lower odds for early AMD (OR = 0.54; 95% CI, 0.33–0.88). Women whose diet scores were in the highest quintile, compared to the lowest, had diets significantly lower in fat (as a percent of energy) and diets higher in median servings of fish, vegetables, dairy, grains and meats or alternatives. The higher quintiles compared to the lower also had more physical activity and fewer years of smoking, lower likelihood of hypertension, lower systolic blood pressure, lower BMI and lower level of serum C-reactive protein. Furthermore, women who had a HLS score of six, which reflected the healthiest of three score components (smoking, physical activity and diet), had 71% lower odds for early AMD compared to those with scores 0–2 [137].

## 11. Conclusions

The proportion of the aged in many societies is growing exponentially. This imposes huge compromises to the public health budgets that must advance quality of life and avoid medical costs for these elderly citizens. Effective means to prevent progress to advanced AMD are clearly crucial toward this end. It appears that a diet that is regularly rich in fruits and vegetables, with sufficient fish, supports good retina health. Supplementation should be considered in the absence of sufficient regular dietary supplies of omega-3 fatty acids, lower glycemic index diets and several micronutrients. Overall healthy lifestyles, including diet, appear to also be beneficial for AMD. The results of the AREDS II trial will inform about the utility of nutrients, e.g., lutein, zeaxanthin, beta carotene and zinc with regard to retinal function.
